# Photothermal Enhancement of Prussian Blue Cathodes for Li-Ion Batteries

**DOI:** 10.1021/acs.nanolett.4c00752

**Published:** 2024-07-19

**Authors:** Lifu Tan, Byung-Man Kim, Arvind Pujari, Ze He, Buddha Deka Boruah, Michael De Volder

**Affiliations:** † Institute for Manufacturing, Department of Engineering, University of Cambridge, Cambridge CB3 0FS, U.K.; ‡ Cambridge Graphene Centre, University of Cambridge, Cambridge CB3 0FA, U.K.; § Cavendish Laboratory, Department of Physics, University of Cambridge, Cambridge CB3 0HE, U.K.; ∥ Institute for Materials Discovery, University College London, London WC1E 7JE, U.K.

**Keywords:** lithium-ion battery, photothermal enhancement, electrochemical impedance spectroscopy, light-enhanced electrochemical reactions, renewable energy technologies, hybrid energy storage, Prussian blue analogues

## Abstract

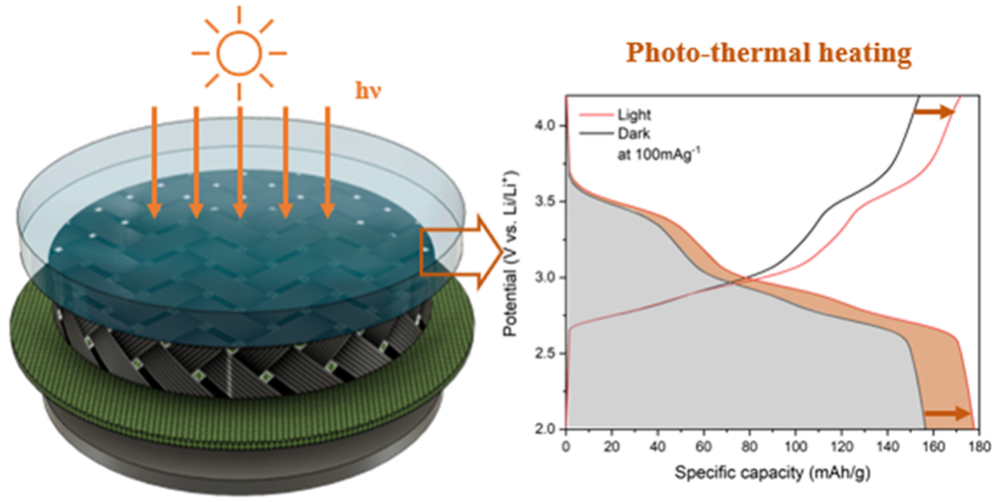

Photoenhanced batteries, where light improves the electrochemical performance of batteries, have gained much interest. Recent reports suggest that light-to-heat conversion can also play an important role. In this work, we study Prussian blue analogues (PBAs), which are known to have a high photothermal heating efficiency and can be used as cathodes for Li-ion batteries. PBAs were synthesized directly on a carbon collector electrode and tested under different thermally controlled conditions to show the effect of photothermal heating on battery performance. Our PBA electrodes reach temperatures that are 14% higher than reference electrodes using a blue LED, and a capacity enhancement of 38% was achieved at a current density of 1600 mA g^–1^. Additionally, these batteries show excellent cycling stability with a capacity retention of 96.6% in dark conditions and 94.8% in light over 100 cycles. Overall, this work shows new insights into the effects leading to improved battery performance in photobatteries.

Recently, there has been an increased interest in studying the effect of shining light on battery electrodes with the view to either increase performance metrics such as the rate capability or capacity or to directly harvest light energy and store it.^1−3^ However, shining light on these photobatteries changes their temperature, and recent work has shown that the effect of heating on electrochemical enhancement has been underestimated.^4^ If one’s goal is to increase battery rate performance or capacity using light, then maximizing the light-induced heat generation is a suitable strategy. In this paper, we leverage the fact that some Li-ion battery (LIB) cathode materials are known to have a high photothermal heating efficiency.^5^ Prussian blue analogues (PBAs) have gained substantial attention in rechargeable batteries. Notably, these materials exhibit a specific capacity of 170 mAh g^–1^ for the Prussian white Na_2_FeFe(CN)_6_ in sodium-ion batteries and 190 mAh g^–1^ for Li_2_FeFe(CN)_6_ in Li-ion batteries.^6−8^ In addition, several studies have shown that PBA analogues are efficient at photothermal heating, arising from their ability to absorb light in the visible or near-infrared (NIR) spectrum efficiently and convert that absorbed energy into nonradiative heat as a result of electronic transitions within the structure. Because of this, PBA nanoparticles have found applications in biomedical applications, such as photothermal therapy for cancer treatment.^9−13^ A very recent paper also reported a Prussian blue solid-polymer-based electrolyte in a solid-state LIB, where photothermal effects are used to improve Li ion diffusion.^14^

In this paper, we seek to leverage the fact that PBA can be used as an LIB cathode and at the same time be heated efficiently using a photothermal effect. These temperature changes are monitored by a combination of electrochemical impedance spectroscopy (EIS) and thermocouples. Under illuminated conditions, we observe temperature increases, reductions in the cell impedance, and improvements in the charging rate of the battery. Furthermore, when the battery was cycled under illumination, the charge/discharge capacity was increased by 14.0% and 37.9% at specific currents of 100 and 1600 mA g^–1^ respectively. These combined measurements show how photothermal heating can be leveraged to increase the electrochemical performance of PBA cathodes in LIBs.

As depicted in Figure 1a, our photothermal LIB cells comprise a window that allows light to reach the PBA coated on carbon felt. Here, Prussian blue serves as the active material, while carbon felt functions as the current collector. In our experiments, we use CR2032 coin cells in which a hole has been drilled, and a window is mounted using a process described previously (see Figure S1).^15^ The carbon felt current collector is connected to the CR2032 cap and provides electric conductivity in the region where the glass window is located, and it also allows for ion transport because of its porous structure that is filled with electrolytes.

**Figure 1:**
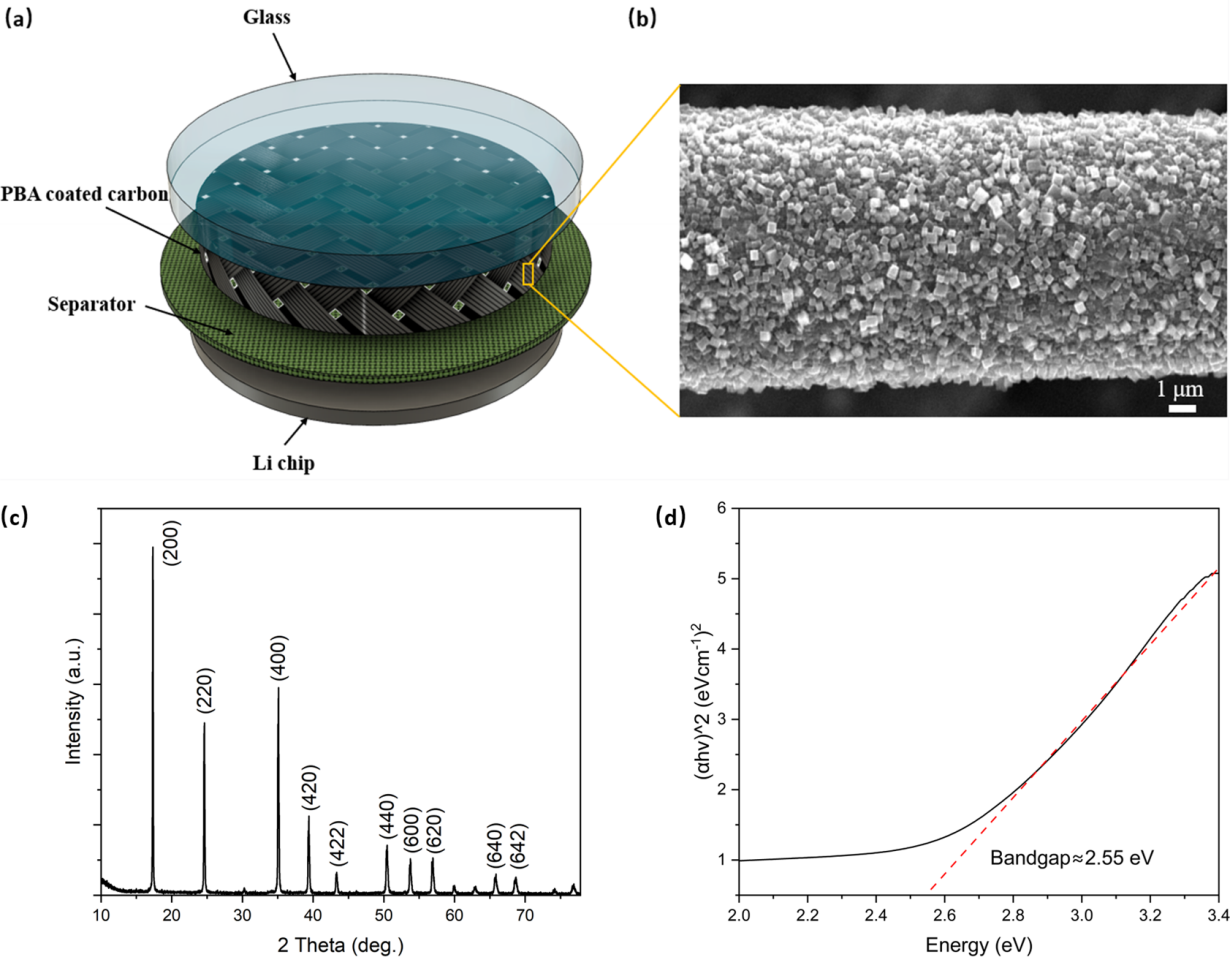
(a) Schematic of a photothermally enhanced PBA/Li half-cell. (b) SEM image of solution-grown Prussian blue on CF as the photocathode. (c) XRD pattern. (d) Determination of the bandgap of ∼2.55 eV by using a Tauc plot.

The PBA was synthesized on the carbon current collector by a hydrolytic precipitation method using K_3_Fe(CN)_6_ as a single iron source (see the Experimental Section in the Supporting Information). Distinct from the rapid coprecipitation method, this approach allows for a uniform coating of nanosized Prussian blue cubes of about 400 to 600 nm on the carbon felt, as shown in Figures 1b and S2.^16,17^ The X-ray diffraction (XRD) plot of Prussian blue is presented in Figure 1c, revealing a face-centered-cubic (fcc) phase ( space group *Fm*3̅*m*).^18^Figure 1d displays the Tauc plot derived from the UV–vis spectrum in Figure S3, showing a bandgap of approximately 2.55 eV, which is similar to the literature value of 2.62 eV.^19^

Before testing our PBA in a battery cell, we verified its optical response as a photodetector using a planar interdigitated electrode configuration, as shown in Figure S4. Unlike batteries, these photodetectors can be operated without any carbon additive, which we used to ensure that the photothermal properties of PBA itself are probed rather than those of carbon additives. Under different bias voltages, we systematically observed an increase in response current when the PBA photodetector was illuminated (see Figure S4). In Figure S4b, the observed rise in maximum current with an elevated bias of the device is noteworthy, and it occurs without any noticeable shift in the curve, unlike the photocarrier generation. As a result, this effect is solely due to heat generation in the PBA by photothermal effects, and this is the first indication that the PBAs are a suitable material for studying photothermal effects in batteries.

To explore changes in the electrochemical behavior of the PBA photocathodes, we first measured cyclic voltammograms in light and dark conditions at scan rates ranging from 0.1 to 1.0 mV s^–1^ over the potential window of 2.0 to 4.2 V. The light source employed had a wavelength of 470 nm with an intensity of 128 mW cm^–2^. As depicted in Figure 2a, two distinct cathodic peaks labeled Peak 1C and Peak 2C are evident at approximately 3.5 and 2.9 V, respectively. Additionally, two corresponding anodic peaks labeled as Peak 1A and Peak 2A are observed at around 3.0 and 3.7 V, respectively. These peaks correspond to subsequent two-step Li^+^ insertion reactions reported previously in the literature:^16^
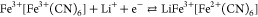
1
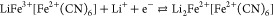
2

**Figure 2:**
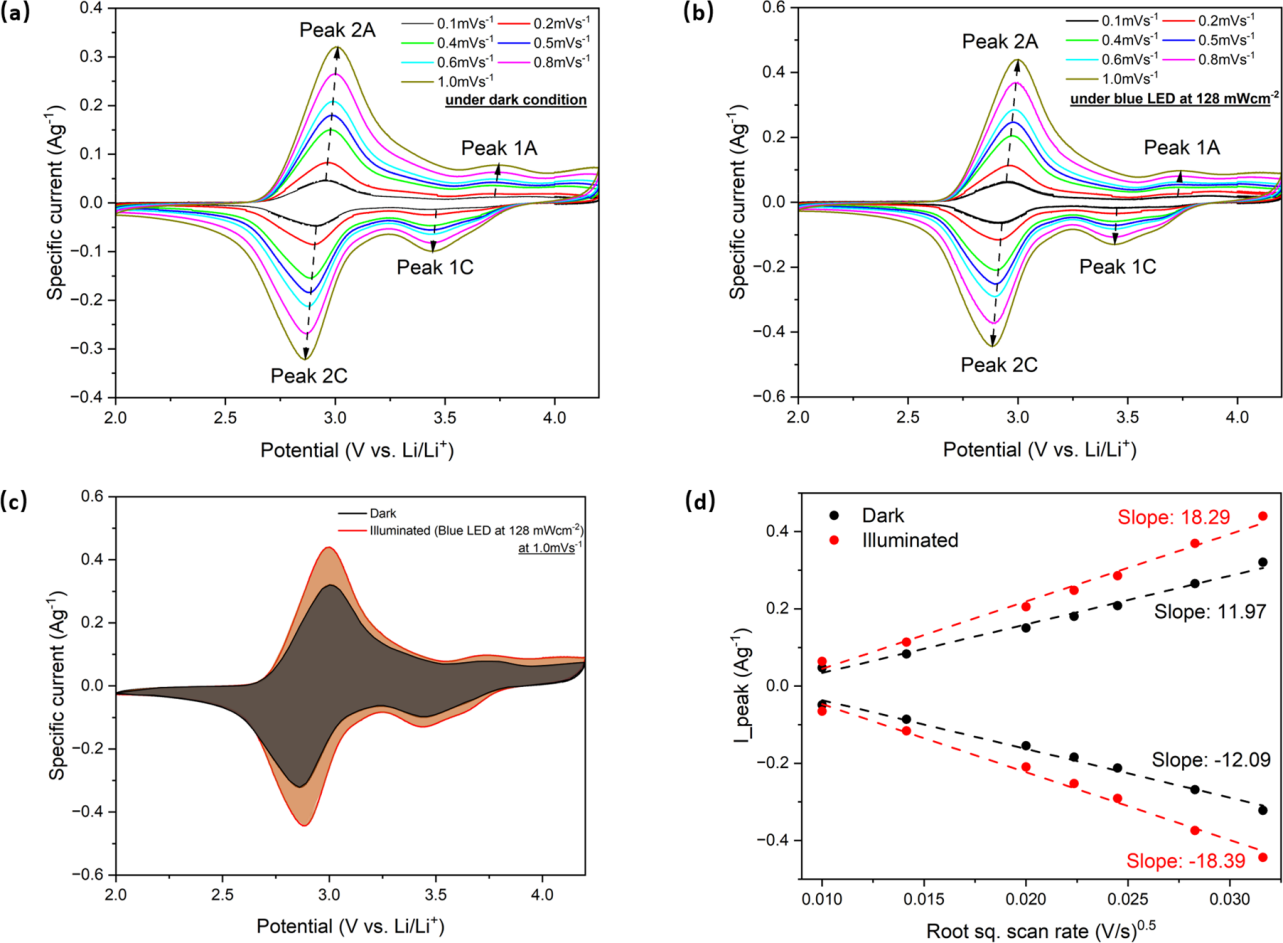
(a, b) CV curves at different scan rates (0.1 to 1.0 mV s^–1^) between 2.0 and 4.2 V in both dark and illuminated conditions. (c) CV curves at 1.0 mV^–1^ in dark and illuminated conditions. (d) Diffusion constant analysis in dark and illuminated conditions.

In comparison to the dark condition, both cathodic peaks and anodic peaks exhibit slight shifts to higher and lower voltages, respectively, in the illuminated cyclic voltammetry (CV) curve, as illustrated in Figure 2b. The shifts are observed from 2.86 to 2.89 V for the 2C cathodic peaks and from 3.01 to 2.99 V for the 2A anodic peaks. By calculating the total sweep area of the CV curves, an ∼28.7% enhancement of the total area is observed in illuminated conditions with a 1.0 mV s^–1^ scan rate, as shown in Figure 2c. Similar trends are evident at different scan rates of 0.4, 0.5, 0.6, and 0.8 mV s^–1^ (see Figure S5). The Li^+^ diffusion constant under both dark and illuminated conditions was investigated by utilizing the peak current densities at the cathodic peak of ∼2.9 V and the anodic peak of ∼3.00 V, spanning a scan rate range from 0.1 to 1.0 mV s^–1^. The Li^+^ diffusion constant (*D*) was calculated using the formula provided below:^20^
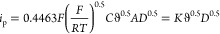
in which
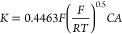
where *i*_p_, *F*, *T*, *C*, ϑ, *A*, and *D* represent the peak current, Faraday constant, absolute temperature, initial concentration in mol cm^–3^, scan rate in V s^–1^, electrode area in cm^2^, and diffusion constant in cm^2^ s^–1^, respectively. Based on linear fits of the slopes of plots of peak current versus the square root of the scan rate under both dark and illuminated conditions, as shown in Figure 2d, the Li^+^ diffusion constants exhibited an increase of ∼52% under light for both anodic and cathodic reactions.

Next, we carried out galvanostatic cycling experiments using specific current densities ranging from 100 to 1600 mA g^–1^ under both dark and illuminated conditions. It was observed that under illumination, the specific capacity increased from 161.0 to 183.5 mAh g^–1^, representing a 14% increment, at a specific current density of 100 mA g^–1^, as shown in Figure 3a. At a specific higher current density of 1600 mA g^–1^, the capacity rose from 99.8 to 137.6 mAh g^–1^, indicating a substantial 38% increment, as illustrated in Figure 3b. The galvanostatic discharge–charge capacities at the specific current densities of 200, 400, and 800 mA g^–1^ under both dark and illuminated conditions are provided in Figure S6. Figure 3c shows the summary of the GCD tests, our Photo-LIBs demonstrate remarkable rate performance and stability under both dark and illuminated conditions. EIS measurements were also performed from 10 mHz to 100 kHz with a voltage amplitude of 10 mV at 50% state of charge (SOC), as illustrated in Figure 3d. Under illumination, the measurements showed a 65% reduction in charge transfer resistance, which decreased from 715 to 250 Ω. The equivalent circuit for the EIS plot can be found in Figure S7. The post-mortem SEM images of photocathodes after lithiation and delithiation (Figure S8) show that PBA remains attached to the carbon fiber current collector.

**Figure 3:**
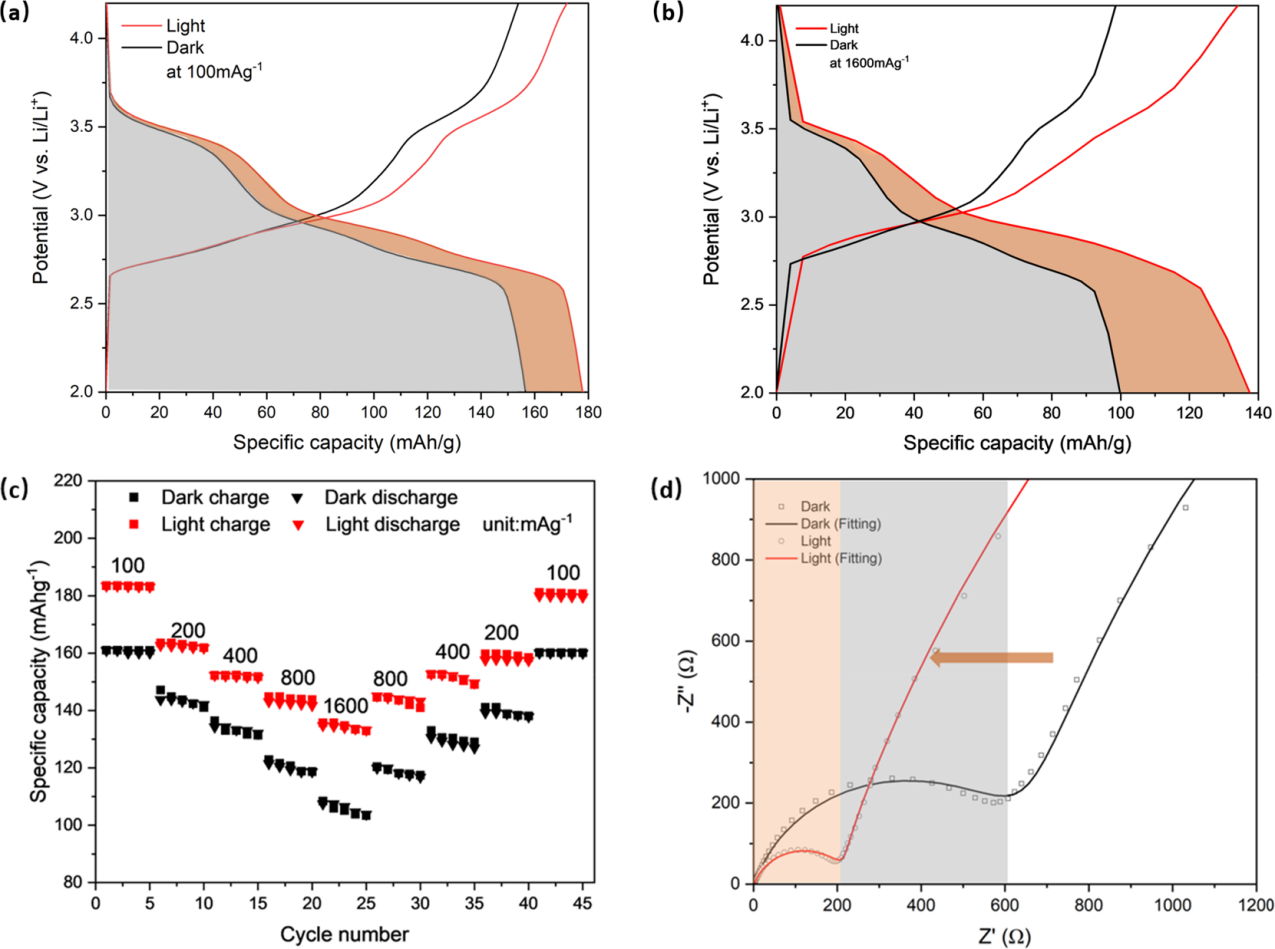
(a) Galvanostatic discharge–charge curves at 100 mA g^–1^ in dark and illuminated conditions. (b) Galvanostatic discharge–charge curves at 1600 mA g^–1^ in dark and illuminated conditions. (c) Rate performance tests of the photo-LIBs in dark and illuminated conditions. (d) EIS measurement of a photo-LIB obtained after the second galvanostatic discharge cycle to 3.0 V (50% SOC) in the frequency range of 10 mHz to 100 kHz at 10 mV amplitude under dark and illuminated conditions.

Next, we analyze the effect of light on the cell temperature. It is challenging to embed temperature probes inside batteries, and therefore, we implemented an impedance-based temperature measurement method.^21^ In this approach, we first let a battery equilibrate in a climate chamber at temperatures ranging from 18 to 38 °C, and subsequently, we carried out EIS measurements at 50% SOC. As shown in Figure 4a, the real part of the impedance at 100 kHz (log) increases with increasing inverse of temperature in a roughly linear trend, as reported in previous studies.^21^ The fitting equation is described as
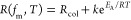
where *k* is the pre-exponential constant factor, *E*_A_ is the molar activation energy, *R* is the gas constant, and *R*_col_ is the resistance of the collector. This equation consists of a typical Arrhenius characteristic part and a constant resistance for the cell parts.^22^ It should be noted that lower frequencies tend to show complicated dependencies on SOC and were therefore avoided in this analysis.^21,23,24^ Next, we carried out the same impedance-based analysis under illumination at different light intensities and converted the 100 kHz impedance (see Figure S9b) to temperature using the calibration curve (Figure S9a), resulting in Figure 4b, which shows linearity within the limited temperature window tested.

**Figure 4:**
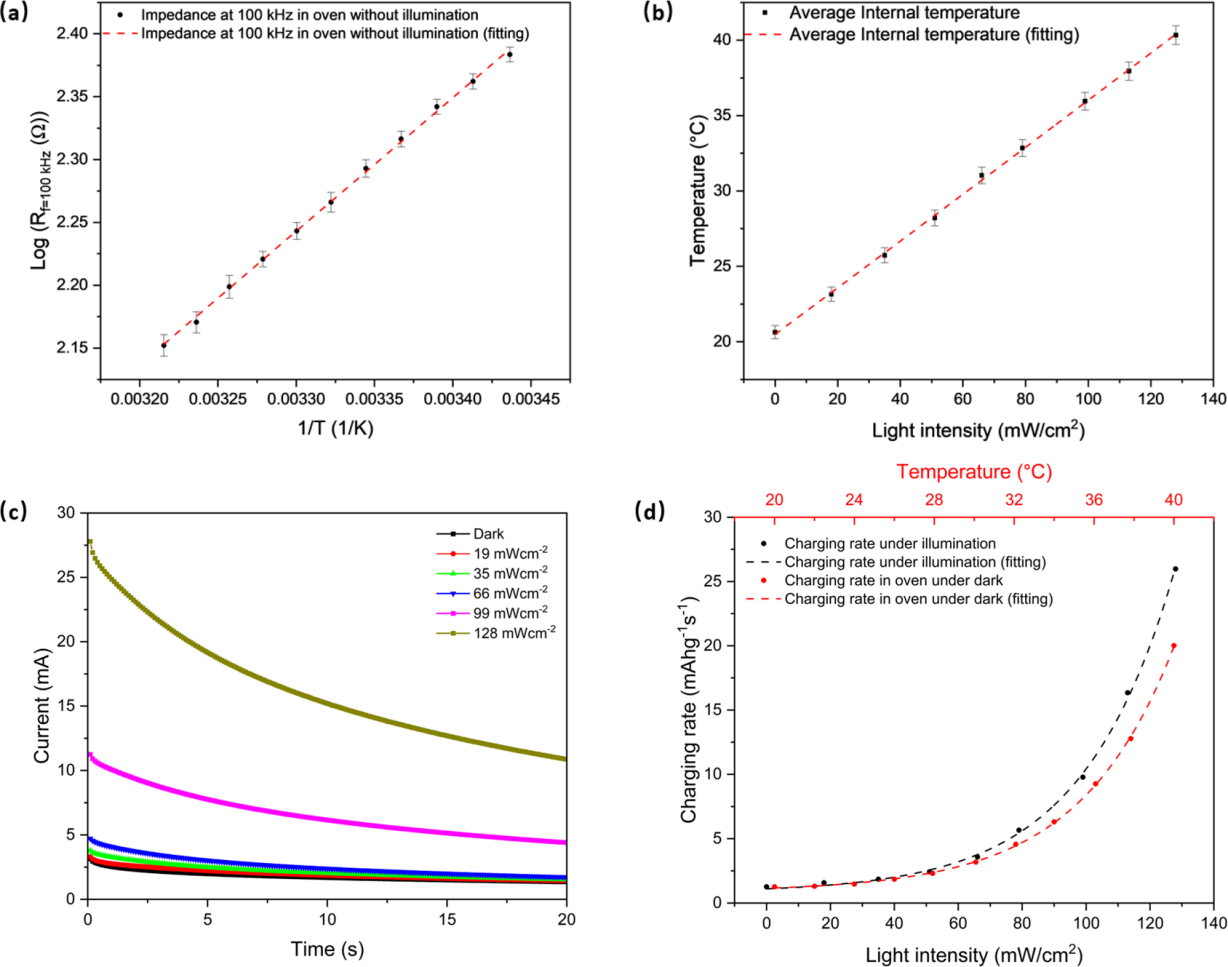
(a) Real part of the impedance at 100 kHz (log) as a function of the inverse of oven temperature with no illumination. (b) Average internal temperature from EIS as a function of light intensity (blue LED). (c) Chronoamperometry curves at different light intensities (blue LED) during constant-voltage-hold charging at 3.2 V vs Li/Li^+^. (d) Charging rate calculated from integration of chronoamperometry curves as a function of light intensity (blue LED) and estimated internal temperature fitted using the Arrhenius law.

Chronoamperometry tests were then conducted at various light intensities, as illustrated in Figure 4c. In these measurements, the cells were fully discharged to 2.0 V. Subsequently, a constant charging voltage of 3.2 V (∼50% SOC) was applied to the cells while the charging current was measured under light with different intensities. With an increase in light intensity, a higher initial charging current was observed (Figure 4c), which is in agreement with the lower impedance observed above under illumination. A very similar increase in current was observed with increasing temperature, as shown in Figure S10. These increases in charge rate under illumination can be compared by integrating the area below the current–time curve and dividing it by the time interval and mass loading. As depicted in Figure 4d, the charging rate exhibits an exponential increase with temperature, in agreement with Arrhenius-type behavior. The very similar changes in chronoamperometry under elevated temperature and illumination again suggest that photothermal heating is a dominating effect in these cells.^25^ The small differences between the temperature and illuminated cells could be due to measurement errors as well as localized inhomogeneous cell temperature or other light-induced effects.

Next, we compare galvanostatic tests at different temperatures carried out in temperature-controlled incubators with the tests described above carried out under different light intensities. Both charge and discharge capacity rise with increasing temperature, as shown in Figure S11. The batteries tested with a 128 mW cm^–2^ blue LED at 40 °C (i.e., the corresponding temperature based on EIS measurements) (Figure 5a) show a similar increase in capacity (difference less than 10 mAh g^–1^). To further elucidate the photothermal effect, we illuminated the cell with a red (630 nm) and a blue (470 nm) LED, both with an intensity of approximately 130 mW cm^–2^. The spectra of both light sources are shown in Figure S13. As shown in Figure 5b, the specific capacity when using a red LED was 11.6% lower compared to the blue LED. This may correspond to the blue LED being above the bandgap energy, leading to more efficient photothermal heating compared to using a red LED. To compare the effect of light wavelength on the temperature increase, we estimated the internal temperature by using EIS measurement, as shown in Figure 5c. This again shows a higher increase in temperature using photothermal heating compared to using the red LED (the impedance at 100 kHz as a function of red LED light intensity and temperature is shown in Figure S12). Next, we measured the increase in temperature during a light-on/off cycle by continuously measuring the impedance at 100 kHz, as shown in Figure S14, which we then converted to temperature as shown in Figure 5d. This further confirms that PBA electrodes reach higher temperatures under blue LEDs. In order to further understand the importance of PB regarding the photothermal conversion, a UV–vis spectrometer with an integrating sphere was used to measure the diffuse-reflectance spectra for bare CF and CF/PBA electrodes, as shown in Figure S15a. This shows that CF with Prussian blue nanoparticles reflects less light than bare CF in the UV–vis range tested here. The temperature increases under light for CF and CF/PB electrodes were also measured in air using a thermocouple to further validate the impedance-based method discussed above, and as shown in Figure S16, these measurements are consistent with the discussion above. Overall, these results indicate the importance of photothermal heating on light-enhanced batteries. Photocharging and photothermal effects can easily be confounded, and more work is needed to unravel these contributions in photobatteries.

**Figure 5:**
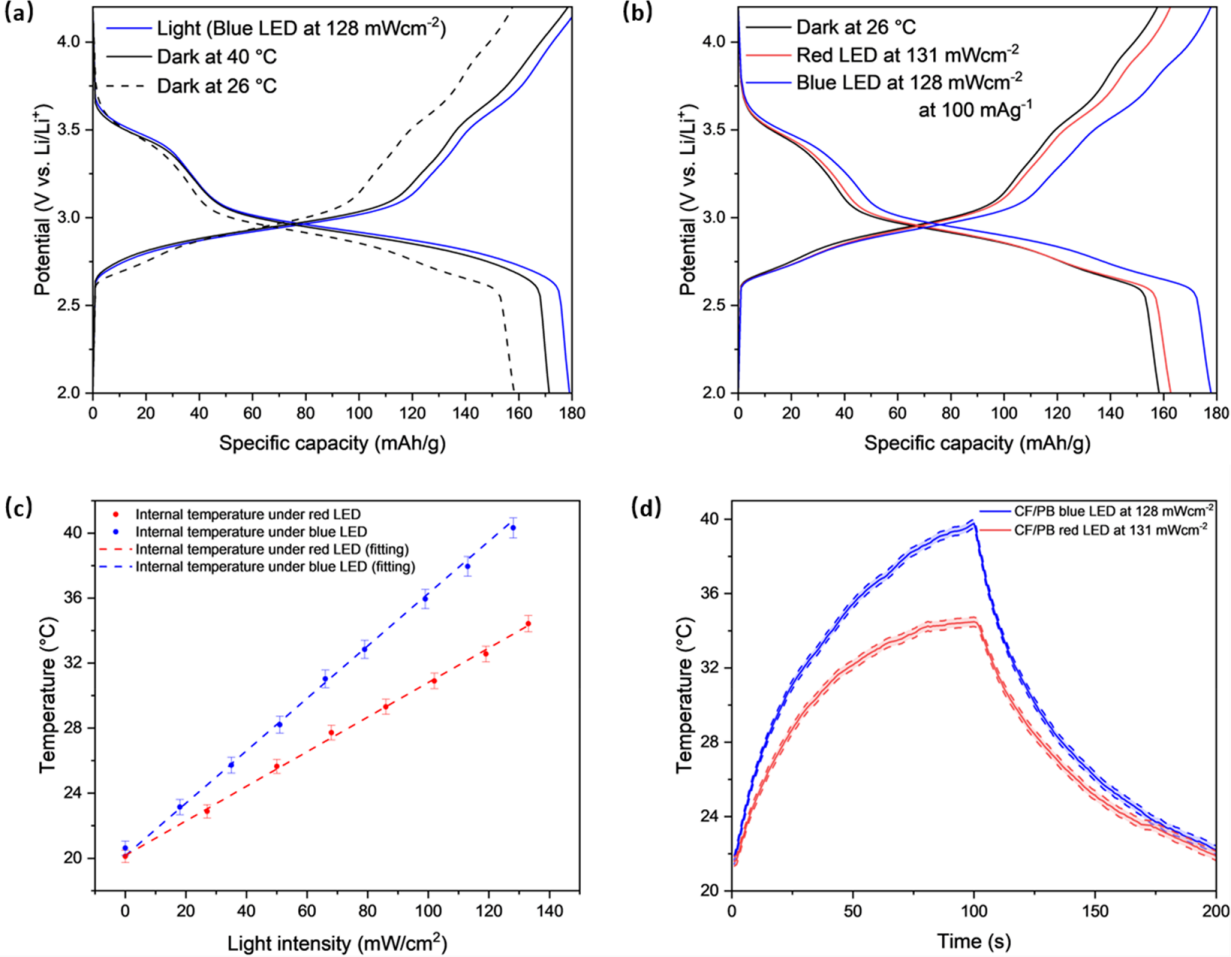
(a) Galvanostatic discharge–charge curves at 100 mA g^–1^ under dark, under a blue LED with a light intensity of 128 mW cm^–2^, and under dark with the corresponding temperature derived from the EIS measurement. (b) Galvanostatic discharge–charge curves at 100 mA g^–1^ under blue and red LEDs. (c) Estimated internal temperature from EIS measurements as a function of light intensity for both red and blue LED. (d) Heating and cooling curves of CF/PB under blue and red LEDs.

To investigate the effect of photothermal heating on the lifetime of our batteries, we cycled three PBA cells for 100 cycles (200 mA g^–1^) at room temperature, at 40 °C, and under illumination (blue LED, 128 mW cm^–2^), and we observed similar capacity retentions of 96.6%, 95.4%, and 94.8%, respectively (see Figures S17–S19). Some of the poor initial Coulombic efficiency of the cycling performance could be due to side reactions in carbon felt.^26^ The difference in nominal charge and discharge voltage (polarization) of the cells over 100 cycles is shown in Figure S20. The results show that heating the cell reduces the cell impedance, as expected from our EIS data, but they also show that over time the polarization in the cell under illumination grows faster (total increase of 55.6%) than that in the control cell heated to 40 °C (total increase of 39%). The latter suggests that light induces additional degradation processes, which we studied by XPS and XRD post-mortem analysis. As shown in Figures S21 and S22, we did not observe any noticeable differences in PBA cathodes cycled under different conditions, which suggests that the increase in polarization observed during these experiments might be due to light-induced reactions with the electrolyte.

In this work, we studied the photothermal effects of light-enhanced PBA cathodes for Li-ion batteries. PBAs are known to have high photothermal heating efficiency and can be used as a cathode in Li-ion batteries and are therefore an interesting model system to study these effects. We implemented an impedance spectroscopy method to measure the temperature of the cell under illumination and found that photothermal heating yields improvements in battery performance that are consistent with control experiments carried out in a climate chamber. Our PBA electrodes reach temperatures that are 14% higher than reference electrodes using a blue LED, and capacity enhancement of 38% was achieved at a current density of 1600 mA g^–1^. Additionally, we observed good cycling stability, with capacity retentions of 96.6% under dark conditions and 94.8% in light over 100 cycles. The possibility of light-induced degradation processes was further examined through XPS and XRD analysis. Despite no observable differences in cathodes, increased polarization may result from light-induced reactions with the electrolyte, suggesting potential avenues for further study to extend the battery lifespan. Overall, this work contributes to advancing the understanding of the intricate mechanisms governing the interaction between light and battery performance and helps better explain improvements in electrochemical performance observed in photobatteries.

## Supporting Information Available

Schematic diagram of the photobattery (Figure S1); SEM images of carbon felt and PB/CF photocathode at low and high magnifications (Figure S2); UV−vis spectrum of Prussian blue (Figure S3); Schematic diagram and digital images of the Au-PB-Au interdigitated electrode, response current and current−voltage curves of the Au-PB-Au PD in dark and illuminated conditions (Figure S4); CVs of PB/CF photocathode (Figure S5); Galvanostatic discharge−charge curves at other current densities (Figure S6); Equivalent circuit for Nyquist plots for the EIS measurement (Figure S7); SEM images of the PB/CF photocathode after lithiation and delithiation (Figure S8); Impedance at 100 kHz as a function of temperature and light intensity (blue LED) (Figure S9); Chronoamperometry curves at different temperatures during constant-voltage hold charging at 3.2 V vs Li/Li^+^ (Figure S10); Galvanostatic discharge−charge curves at 100 mAg^–1^ at a range of temperatures (Figure S11); Impedance at 100 kHz as a function of light intensity (red LED) and measured oven temperature and estimated internal temperature as a function of light intensity (red LED) (Figure S12); Spectra of the blue and red LEDs (Figure S13); Impedance at 100 kHz during a continuous light on/off cycle for blue LEDs at 128 mW cm^−2^ and red LEDs at 131 mW cm^−2^, impedance at 100 kHz as a function of temperature for the CF/Li half-cell, and impedance at 100 kHz during a continuous light-on/off cycle for the blue LED at 128 mW cm^−2^ for the CF/Li half-cell (Figure S14); Reflectance spectra and heating and cooling curves of bare CF and CF/PB electrodes under blue LED (Figure S15); Heating and cooling curves of CF/PB and bare CF under blue and red LEDs (Figure S16); Galvanostatic charge−discharge curves at the specific current density of 200 mA g^–1^ under dark conditions at 26 °C at selected cycles and long-term cycling stability of the photo-LIBs under dark conditions at 26 °C at 200 mA g^–1^. (Figure S17); Galvanostatic charge−discharge curves at the specific current density of 200 mA g^-1^ under dark conditions at 40 °C at selected cycles and long-term cycling stability of the photo-LIBs under dark conditions at 40 °C at 200 mA g^–1^. (Figure S18); Galvanostatic charge−discharge curves at the specific current density of 200 mA g^–1^ under light conditions at 26 °C at selected cycles and long-term cycling stability of the photo-LIBs under light conditions at 26 °C at 200 mA g^–1^. (Figure S19); Differences between nominal charge and discharge voltage (ΔV) as a function of cycle number under dark at 26 °C, dark at 40 °C, and light at 26 °C (Figure S20); XPS spectra of the characteristic Fe 2p peaks under dark at 26 °C, dark at 40 °C, and light at 26 °C before and after cycling (Figure S21); XRD patterns of PB/CF electrodes under dark at 26 °C, dark at 40 °C, and light at 26 °C before and after cycling (Figure S22) (PDF)

## References

[ref1] Andersen H.; Lu Y.; Borowiec J.; Parkin I. P.; De Volder M.; Deka Boruah B. Photo-enhanced lithium-ion batteries using metal–organic frameworks. Nanoscale 2023, 15 (8), 4000–4005. 10.1039/D3NR00257H. 36723271 PMC9949567

[ref2] Pandya R.; Mathieson A.; Boruah B. D.; de Aguiar H. B.; de Volder M. Interrogating the Light-Induced Charging Mechanism in Li-Ion Batteries Using Operando Optical Microscopy. Nano Lett. 2023, 23 (16), 7288–7296. 10.1021/acs.nanolett.3c01148. 37552026 PMC10450808

[ref3] Park S. K.; Boruah B. D.; Pujari A.; Kim B.-M.; De Volder M. Photo-Enhanced Magnesium-Ion Capacitors Using Photoactive Electrodes. Small 2022, 18 (38), 2202785 10.1002/smll.202202785. 35988148

[ref4] Pujari A.; Kim B.-M.; Sayed F. N.; Sanders K.; Dose W. M.; Mathieson A.; Grey C. P.; Greenham N. C.; De Volder M. Does Heat Play a Role in the Observed Behavior of Aqueous Photobatteries?. ACS Energy Letters 2023, 8 (11), 4625–4633. 10.1021/acsenergylett.3c01627. 37969251 PMC10644369

[ref5] Cui X.; Ruan Q.; Zhuo X.; Xia X.; Hu J.; Fu R.; Li Y.; Wang J.; Xu H. Photothermal Nanomaterials: A Powerful Light-to-Heat Converter. Chem. Rev. 2023, 123 (11), 6891–6952. 10.1021/acs.chemrev.3c00159. 37133878 PMC10273250

[ref6] Zhang Z.; Avdeev M.; Chen H.; Yin W.; Kan W.; He G. Lithiated Prussian blue analogues as positive electrode active materials for stable non-aqueous lithium-ion batteries. Nat. Commun. 2022, 13, 7790 10.1038/s41467-022-35376-1. 36526618 PMC9758126

[ref7] Hurlbutt K.; Wheeler S.; Capone I.; Pasta M. Prussian Blue Analogs as Battery Materials. Joule 2018, 2 (10), 1950–1960. 10.1016/j.joule.2018.07.017.

[ref8] Liu S.; Kang L.; Jun S. C. Challenges and Strategies toward Cathode Materials for Rechargeable Potassium-Ion Batteries. Adv. Mater. 2021, 33 (47), 2004689 10.1002/adma.202004689. 33448099

[ref9] Dacarro G.; Taglietti A.; Pallavicini P. Prussian Blue Nanoparticles as a Versatile Photothermal Tool. Molecules 2018, 23 (6), 1414 10.3390/molecules23061414. 29891819 PMC6099709

[ref10] Hoffman H. A.; Chakrabarti L.; Dumont M. F.; Sandler A. D.; Fernandes R. Prussian blue nanoparticles for laser-induced photothermal therapy of tumors. RSC Adv. 2014, 4 (56), 29729–29734. 10.1039/C4RA05209A.

[ref11] Fu G.; Liu W.; Feng S.; Yue X. Prussian blue nanoparticles operate as a new generation of photothermal ablation agents for cancer therapy. Chem. Commun. 2012, 48 (94), 11567–11569. 10.1039/c2cc36456e. 23090583

[ref12] Li J.; Liu X.; Tan L.; Cui Z.; Yang X.; Liang Y.; Li Z.; Zhu S.; Zheng Y.; Yeung K. W. K.; et al. Zinc-doped Prussian blue enhances photothermal clearance of Staphylococcus aureus and promotes tissue repair in infected wounds. Nat. Commun. 2019, 10 (1), 4490 10.1038/s41467-019-12429-6. 31582736 PMC6776522

[ref13] Gärtner W. W. Photothermal Effect in Semiconductors. Phys. Rev. 1961, 122 (2), 419–424. 10.1103/PhysRev.122.419.

[ref14] Wang Q.; Sun Q.; Pu Y.; Sun W.; Lin C.; Duan X.; Ren X.; Lu L. Photo-Thermal Mediated Li-ion Transport for Solid-State Lithium Metal Batteries. Small 2024, 20 (22), 2309501 10.1002/smll.202309501. 38109067

[ref15] Kato K.; Puthirath A. B.; Mojibpour A.; Miroshnikov M.; Satapathy S.; Thangavel N. K.; Mahankali K.; Dong L.; Arava L. M. R.; John G.; et al. Light-Assisted Rechargeable Lithium Batteries: Organic Molecules for Simultaneous Energy Harvesting and Storage. Nano Lett. 2021, 21 (2), 907–913. 10.1021/acs.nanolett.0c03311. 33416335

[ref16] Wu X.; Shao M.; Wu C.; Qian J.; Cao Y.; Ai X.; Yang H. Low Defect FeFe(CN)_6_ Framework as Stable Host Material for High Performance Li-Ion Batteries. ACS Appl. Mater. Interfaces 2016, 8 (36), 23706–23712. 10.1021/acsami.6b06880. 27556906

[ref17] Wu X.; Deng W.; Qian J.; Cao Y.; Ai X.; Yang H. Single-crystal FeFe(CN)_6_ nanoparticles: a high capacity and high rate cathode for Na-ion batteries. J. Mater. Chem. A 2013, 1 (35), 10130–10134. 10.1039/c3ta12036h.

[ref18] Kumar A.; Yusuf S.; Keller L. Structural and magnetic properties of Fe[Fe(CN)_6_]·4H_2_O. Phys. Rev. B 2005, 71, 54414 10.1103/PhysRevB.71.054414.

[ref19] Hegner F. S.; Galán-Mascarós J. R.; López N. A Database of the Structural and Electronic Properties of Prussian Blue, Prussian White, and Berlin Green Compounds through Density Functional Theory. Inorg. Chem. 2016, 55 (24), 12851–12862. 10.1021/acs.inorgchem.6b02200. 27989203

[ref20] Yu D.; Fietzek C.; Weydanz W.; Donoue K.; Inoue T.; Kurokawa H.; Fujitani S. Study of LiFePO_4_ by Cyclic Voltammetry. J. Electrochem. Soc. 2007, 154, A253–A257. 10.1149/1.2434687.

[ref21] Schmidt J. P.; Arnold S.; Loges A.; Werner D.; Wetzel T.; Ivers-Tiffée E. Measurement of the internal cell temperature via impedance: Evaluation and application of a new method. J. Power Sources 2013, 243, 110–117. 10.1016/j.jpowsour.2013.06.013.

[ref22] Park M.; Zhang X.; Chung M.; Less G. B.; Sastry A. M. A review of conduction phenomena in Li-ion batteries. J. Power Sources 2010, 195 (24), 7904–7929. 10.1016/j.jpowsour.2010.06.060.

[ref23] Abraham D. P.; Kawauchi S.; Dees D. W. Modeling the impedance versus voltage characteristics of LiNi0.8Co0.15Al0.05O2. Electrochim. Acta 2008, 53 (5), 2121–2129. 10.1016/j.electacta.2007.09.018.

[ref24] Wu M.-S.; Chiang P.-C. J.; Lin J.-C. Electrochemical Investigations on Advanced Lithium-Ion Batteries by Three-Electrode Measurements. J. Electrochem. Soc. 2005, 152 (1), A47 10.1149/1.1825385.

[ref25] Baffou G.; Bordacchini I.; Baldi A.; Quidant R. Simple experimental procedures to distinguish photothermal from hot-carrier processes in plasmonics. Light: Sci. Appl. 2020, 9 (1), 108 10.1038/s41377-020-00345-0. 32612818 PMC7321931

[ref26] Pujari A.; Kim B.; Greenham N.; De Volder M. Identifying Current Collectors That Enable Light Battery Interactions. Small Methods 2024, 2301572 10.1002/smtd.202301572. 38695753

