# Supplementary material for: Photothermal Enhancement of Prussian Blue Cathodes for Li-Ion Batteries
Source: Nano Lett. 2024 Jul 19;24(30):9147–54. doi: 10.1021/acs.nanolett.4c00752 (PMC11299224; doi:10.1021/acs.nanolett.4c00752)
Supplement: Supplementary file 1 — nl4c00752_si_001.pdf [file nl4c00752_si_001.pdf]

Supporting information

For

Photo-thermal enhancement of Prussian blue cathodes for Li-ion batteries

Lifu Tan[a,b], Byung-Man Kim[a], Arvind Pujari[a,c], Ze He[a], Buddha Deka Boruah[d], Michael De Volder[a,*]

[a]Institute for Manufacturing, Department of Engineering, University of Cambridge, Cambridge CB3 0FS, UK

[b]Cambridge Graphene Centre, University of Cambridge, Cambridge CB3 0FA, UK

[c]Cavendish Laboratory, Department of Physics, University of Cambridge, Cambridge, CB3 0HE, UK

[d]Institute for Materials Discovery, University College London, London, WC1E 7JE, UK

[*]Corresponding author. E-mail: mfld2@cam.ac.uk

Experimental Section:

Material Synthesis and Photoelectrode Preparation:

The synthesis of Prussian blue type nanocrystals followed a facile hydrolytic precipitation method adapted from the literature. Initially, 20 mmol (6.5844g) of $K_3Fe(CN)_6 \cdot 10H_2O$ was dissolved in 100 ml of deionized water (DI H_2O) and thoroughly stirred. Subsequently, a piece of carbon felt, pre-treated with UV-Ozone to enhance wettability, was introduced into the solution. Following this, 1 ml of 37% hydrochloric acid was added, and the beaker was heated to 60 °C under vigorous stirring. After 24 hours, the carbon felt was removed from the solution and placed into a vacuum oven. Finally, the sample was dried overnight at 60°C. The resulting photocathode was then cut into a 9 mm diameter for the synthesis of photo-LIBs.

Material Characterization:

The morphologies of the Prussian blue material were examined using scanning electron microscopy (Phenom-SEM). X-ray diffraction (XRD) patterns were recorded using a Bruker D8 Advance instrument with Cu K_α radiation and a scan rate of 6° min^{-1}. Optical properties and the bandgap of the material were determined using a PerkinElmer UV/Vis/NIR Spectrometer (Lambda 750). The temperature of the CF and CF/PB electrode was measured by using a THORLABs 2-channel compact USB temperature and humidity data logger. XPS analysis was performed using a Thermo Scientific Escalab 250Xi fitted with a monochromated Al kα X-ray source (1486.7 eV). All data was recorded with an X-ray beam size of 650 µm, a pass energy of 20 eV at a step size of 0.1 eV. Electronic charge neutralization was achieved using an ion source. Ion gun current = 100 µA. Ion gun voltage = 40 V. All sample data was recorded at a pressure below 10^{-8} Torr and a room temperature of 294 K. Data was analysed using CasaXPS v2.3.26rev1.0N.

Design of the Photo-LIB:

To assemble the photo-enhanced lithium-ion battery (photo-LIB), a 10mm diameter hole was drilled in a coin cell (CR2032) can, and a glass window was placed over it. The glass was sealed using epoxy glue. The photocathode was positioned on the glass window, and Aluminium strips were utilized to establish connections between the photocathode and the coin-cell casing. Following this, a piece of Whatman glass microfiber filter paper separator was placed atop the photocathode, and 70 µL of $LiPF_6$ in EC/EMC (1:1) electrolyte was added. The Li metal counter electrode was then positioned on the separator. Finally, the photo-LIB was assembled by adding a spacer and spring on the Li anode side.

Electrochemical Characterization of the Photo-LIB:

The electrochemical measurements of the photo-enhanced lithium-ion batteries (photo-LIBs) were conducted using a Biologic VMP-3 galvanostat. Initially, galvanostatic discharge-charge tests (GCD) were performed at a current density ranging from 100 mAg^{-1} to 1600 mAg^{-1} in both dark and illuminated conditions. The tests were carried out using the designed photo-LIB devices within a working voltage range of 2.0 to 4.2V. Additionally, cyclic voltammetry (CV) tests were recorded at various scan rates, ranging from 0.1mVs^{-1} to 1.0mVs^{-1}, over the potential window of 2.0 to 4.2V under both dark and illuminated conditions (Light source $\lambda \approx 470$ nm, intensity ≈ 128 mW cm^{-2}). Furthermore, AC impedance (EIS) measurements were conducted at a frequency ranging from 10 mHz to 100 kHz, with a voltage amplitude of 10 mV, in both dark and illuminated conditions. Thermal-compared chronoamperometry, EIS and GCD measurements were tested in a closed temperature-controlled incubator at a range of 26 °C to 36 °C. Wavelength-controlled GCD measurement was done by using a red LED. (Light source $\lambda \approx 630$ nm, intensity ≈ 131 mW cm^{-2})

Design of the interdigitated electrode (IDE) and measurement:

The carbon felt containing the synthesized Prussian blue nanocrystals underwent sonication with additional DI water for 30 minutes. Subsequently, the resulting dispersion was drop-cast onto the Au-PB-Au interdigitated electrode and dried on a hot plate at 80 °C for 3 hours. Current-time (I-t) measurements were conducted using a Keysight B2901 source measure unit, with photocurrent recorded under alternating dark and light conditions at an external bias voltage (V= -50 mV to 50 mV).

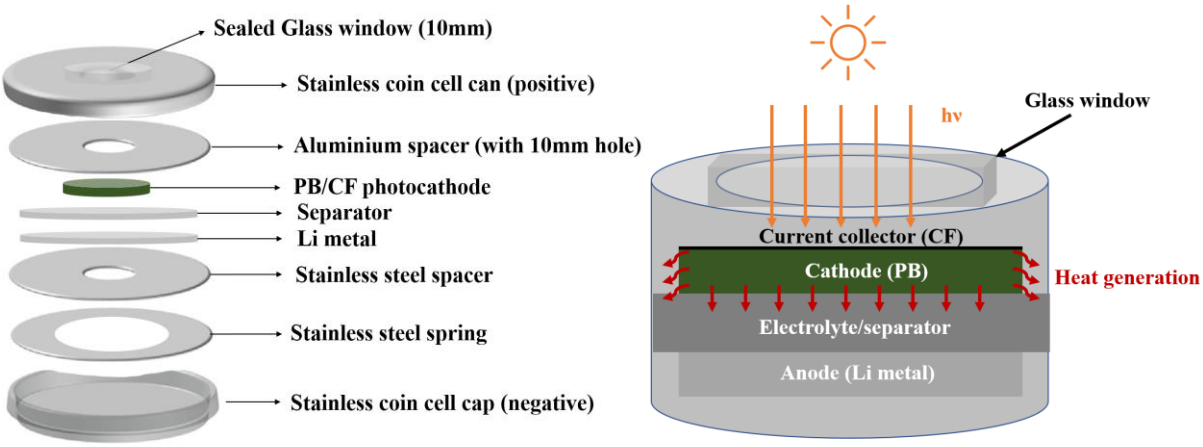

Figure S1. *Schematic diagrams of the photo-battery with PB/CF photocathode and Li foil as the anode.*

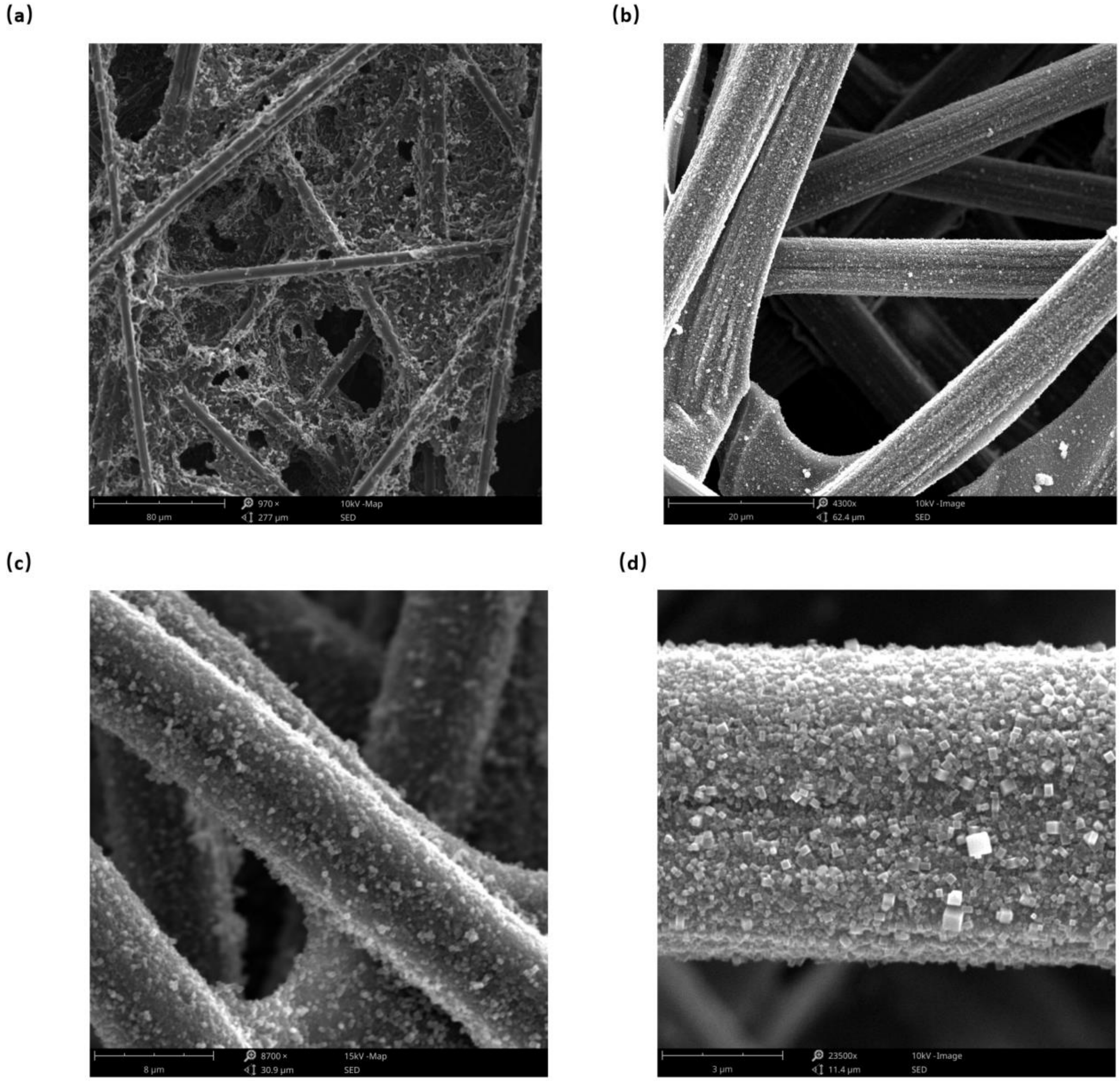

Figure S2. *SEM images of (a) Carbon felt, (b, c, d) PB/CF photocathode at low and high magnifications.*

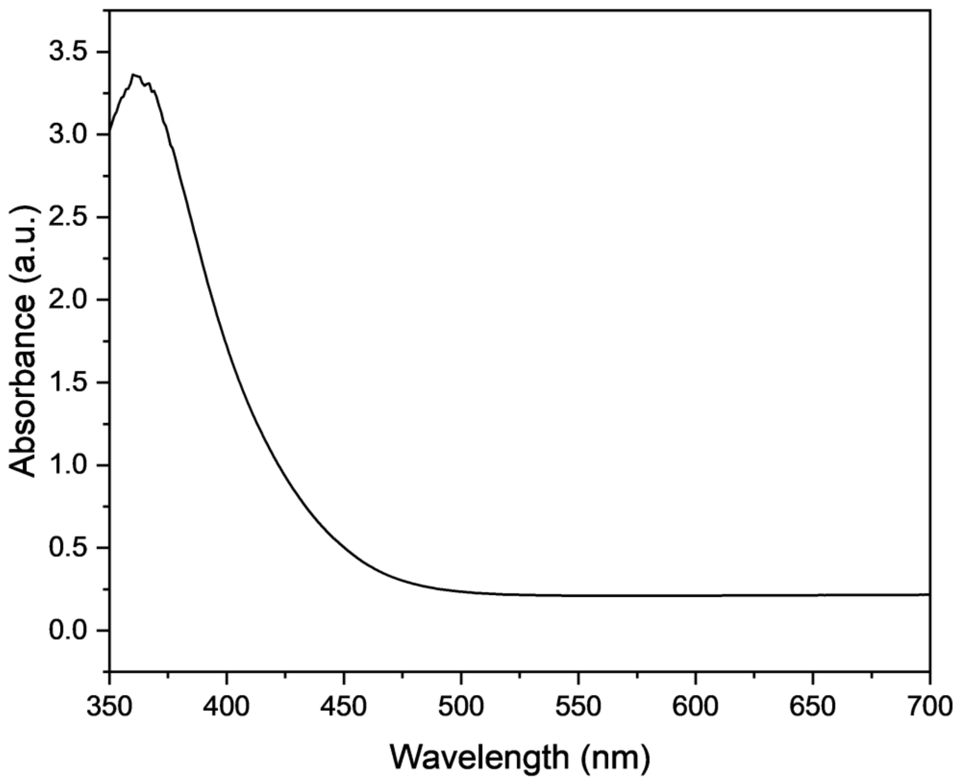

Figure S3. *UV-VIS spectrum of Prussian blue.*

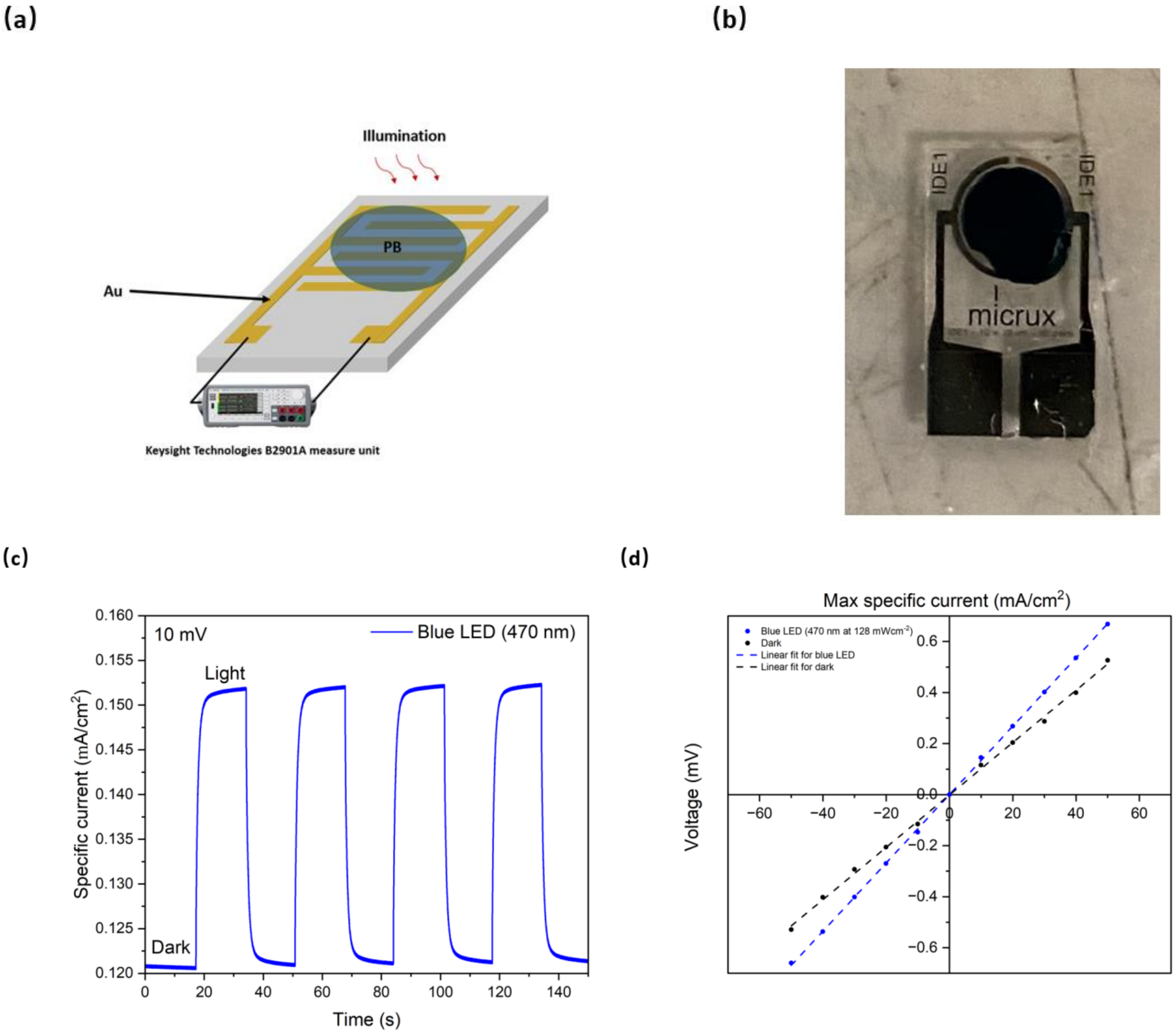

Figure S4. *(a) Schematic diagram of the Au-PB-Au interdigital electrode. (b) Digital images of the Au-PB-Au interdigitated electrode. (c) Response current of the Au-PB-Au PD under alternating dark and illuminated conditions at 10 mV. (d) Current-voltage curves of the planar Au-PB-Au PD in dark and illuminated conditions.*

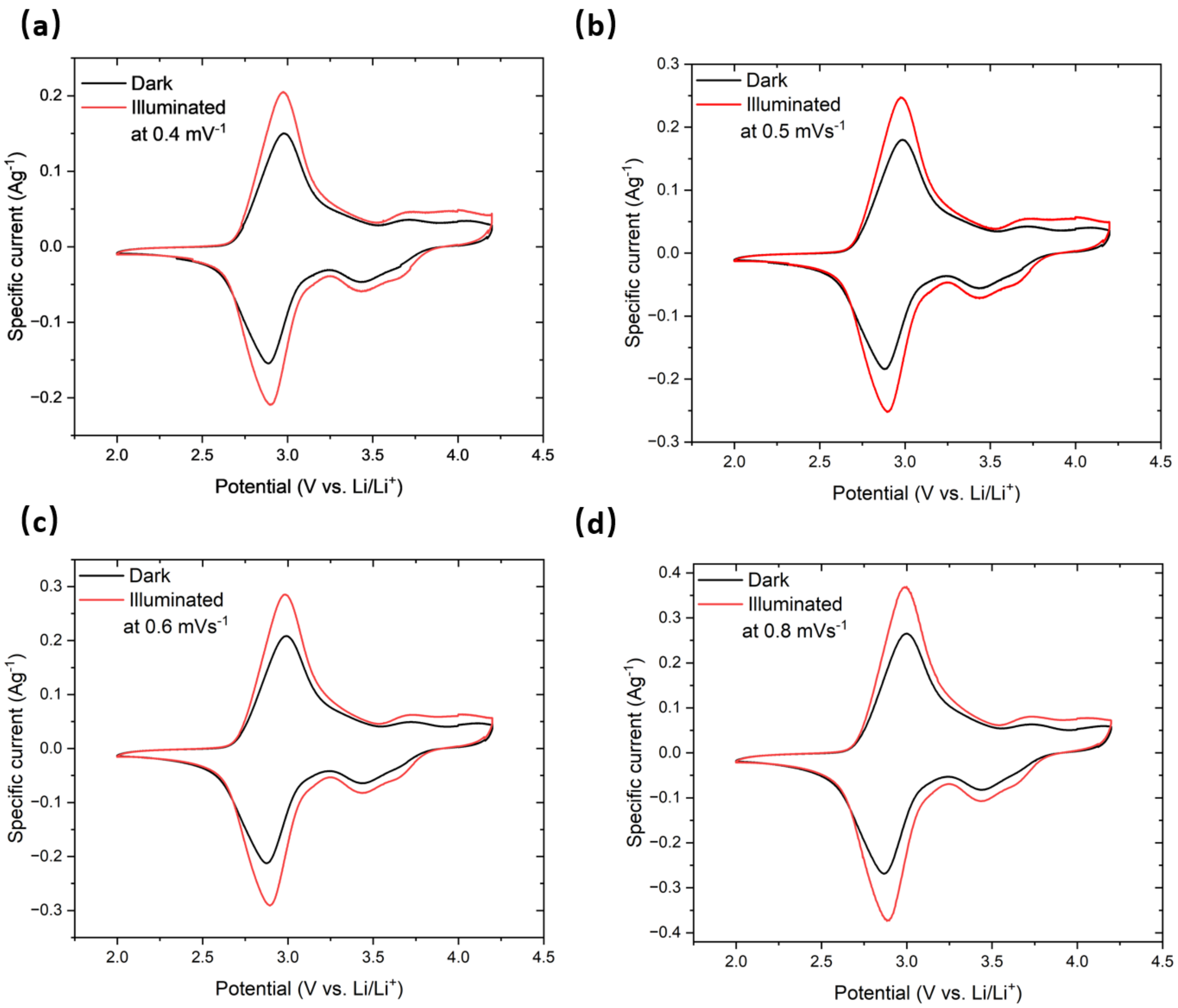

Figure S5. *CV scans at rate of (a) 0.4 mVs^{-1}, (b) 0.5 mVs^{-1}, (c) 0.6 mVs^{-1} and (d) 0.8 mVs^{-1} in dark and illuminated conditions.*

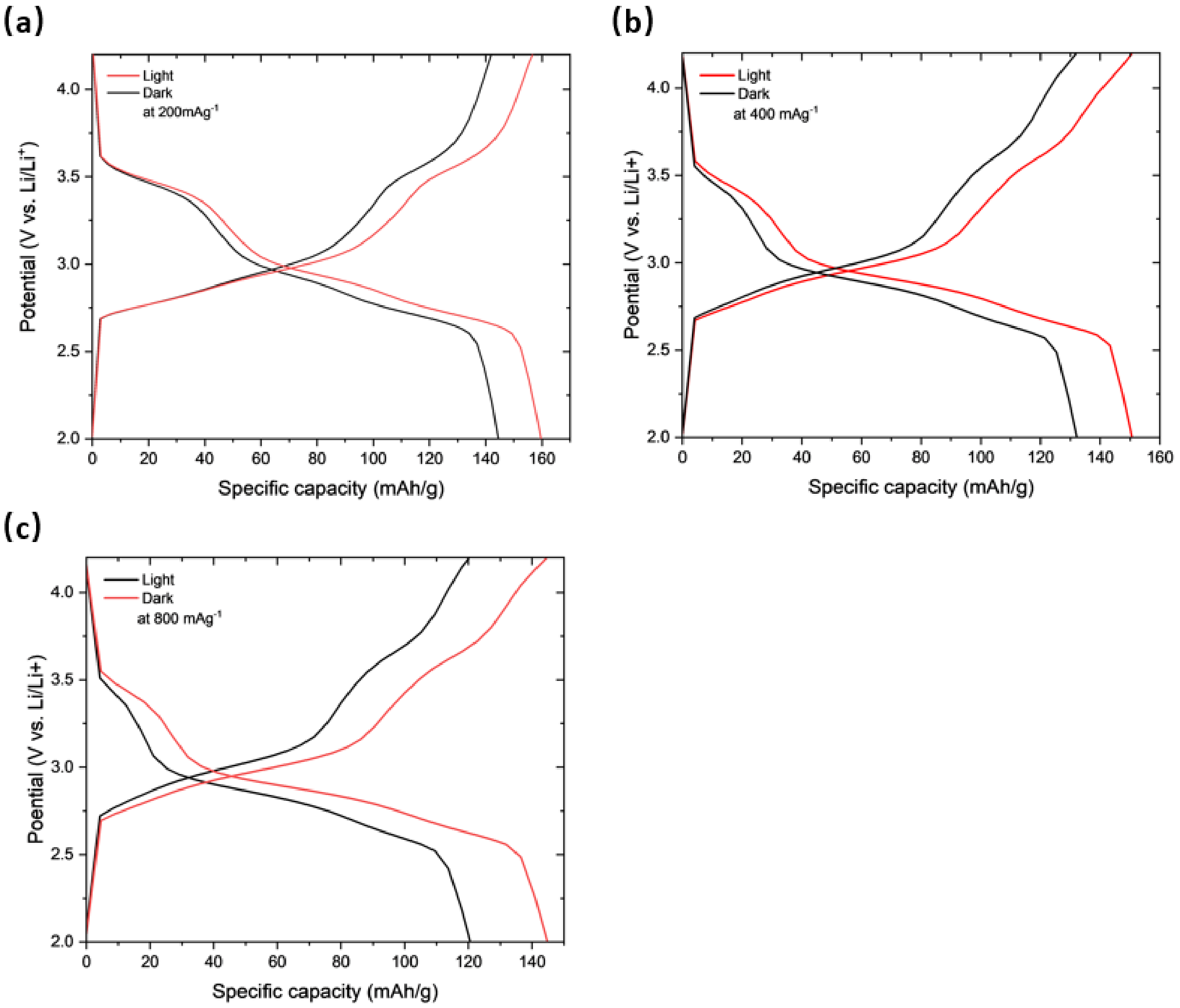

Figure S6. *Galvanostatic charge-discharge curves at the specific current density of (a) 200 mAg*$^{-1}$*, (b) 400 mAg*$^{-1}$ *and (c) 800 mAg*$^{-1}$ *(d) 1600 mAg*$^{-1}$ *under light and dark conditions.*

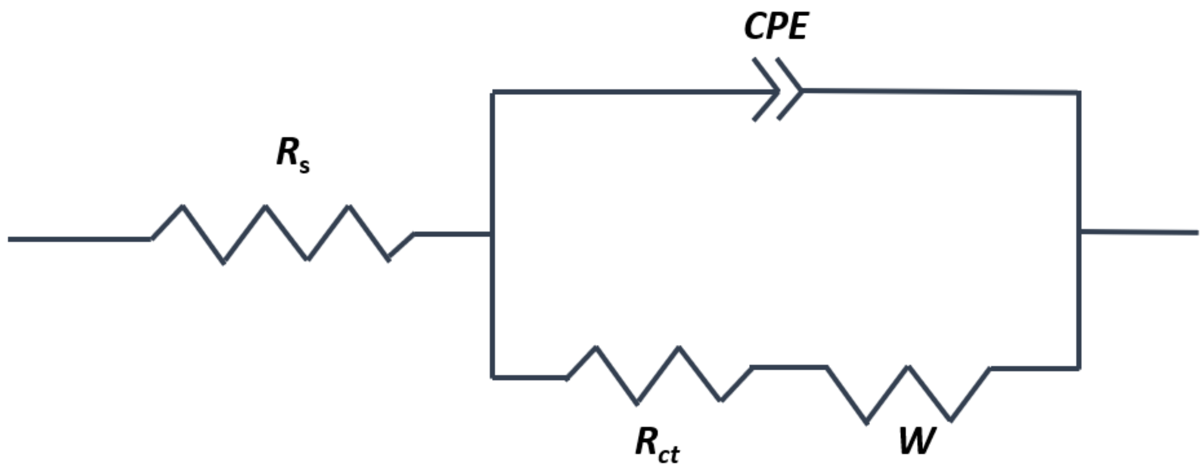

Figure S7. *Equivalent circuit for Nyquist plots for the EIS measurement, where R_s corresponds to the total resistance of the electrode, electrolyte, and separator, R_{ct} refers to the charge transfer resistance, CPE represents constant phase element corresponding to the semicircles, and W refers to the Warburg impedance.*

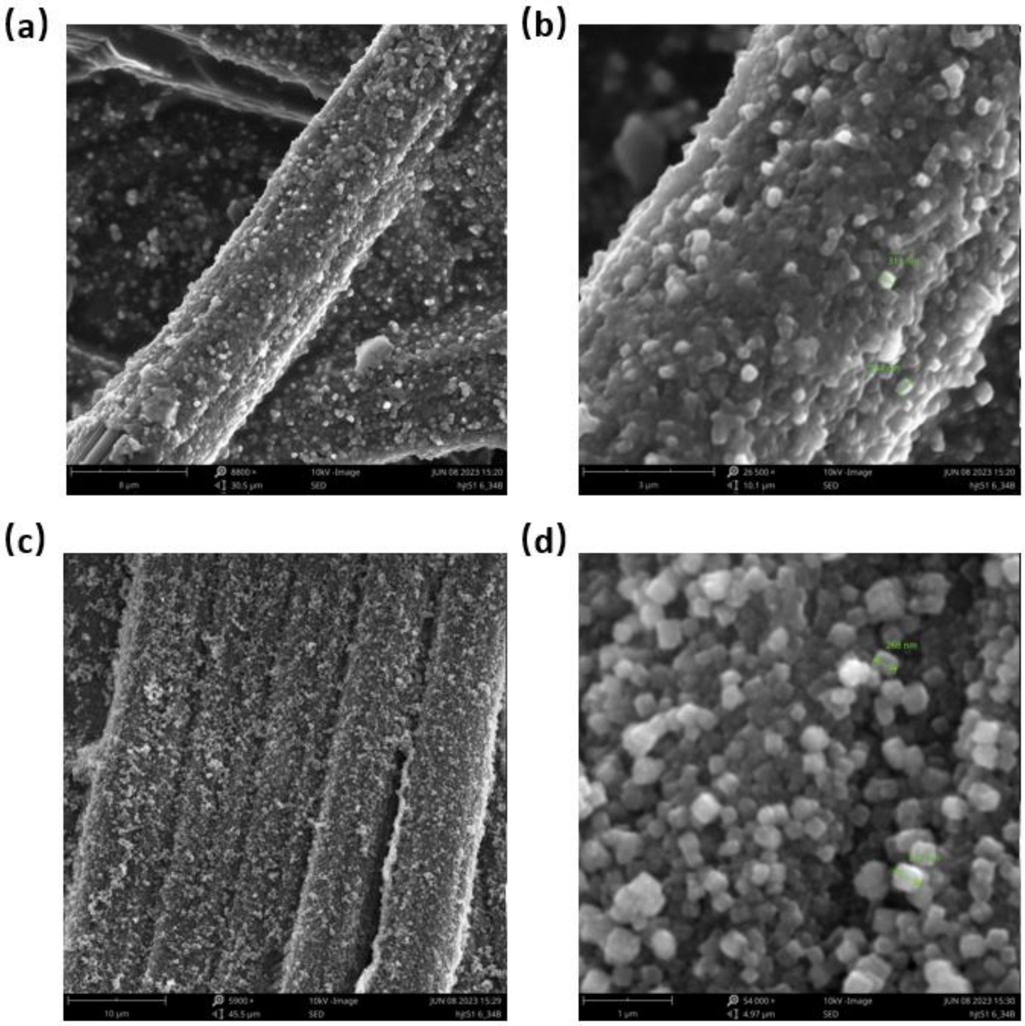

Figure S8. *SEM images of (a, b) photocathode after lithiation (discharged to 2.0 V) at different magnifications (c, d) after de-lithiation (charged to 4.2 V) at different magnifications.*

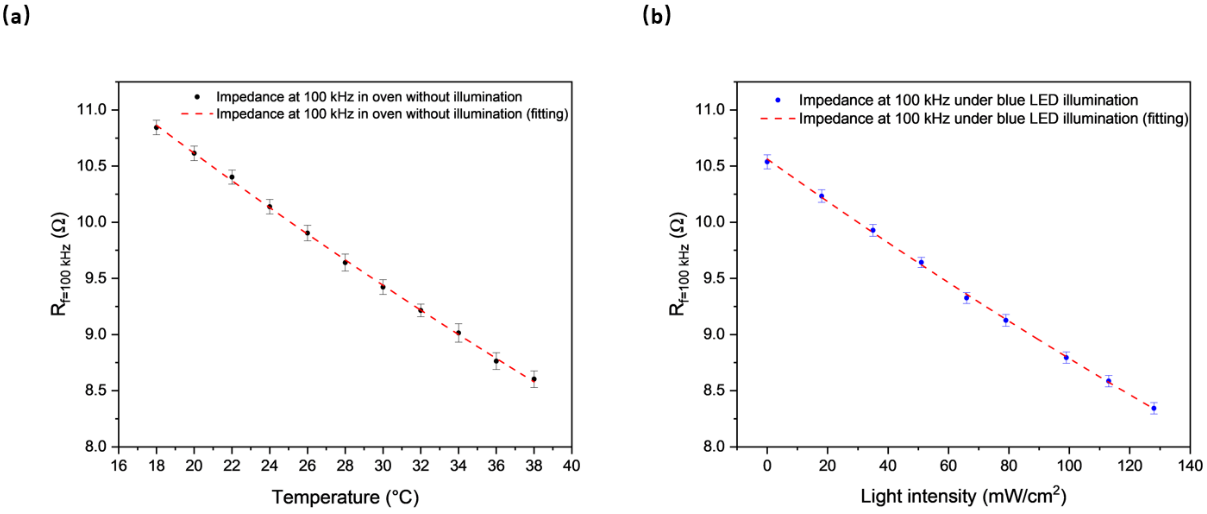

Figure S9. (a) *Impedance at 100 kHz as a function of temperature.* (b) *Impedance at 100 kHz as a function of light intensity (Blue LED). (Fit: Arrhenius + R)*

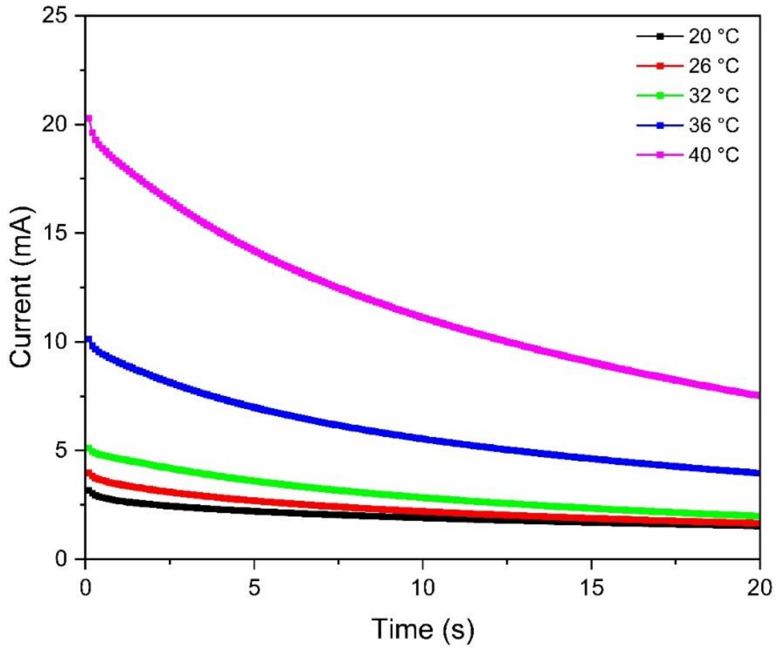

Figure S10. *Chronoamperometry curves at different temperatures during constant voltage hold charging at 3.2 V vs. Li/Li^+.*

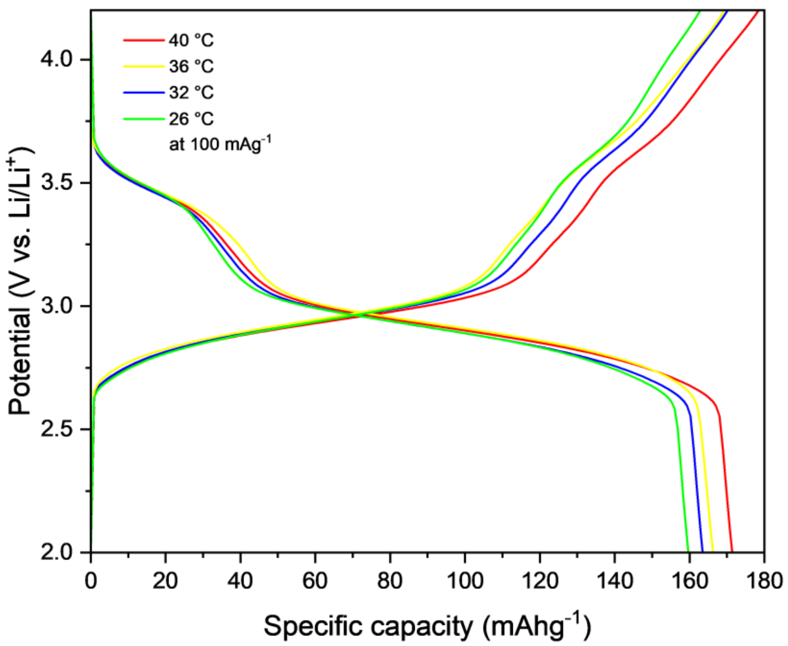

Figure S11. *Galvanostatic discharge-charge curves at 100 mAg^{-1} at a range of temperatures.*

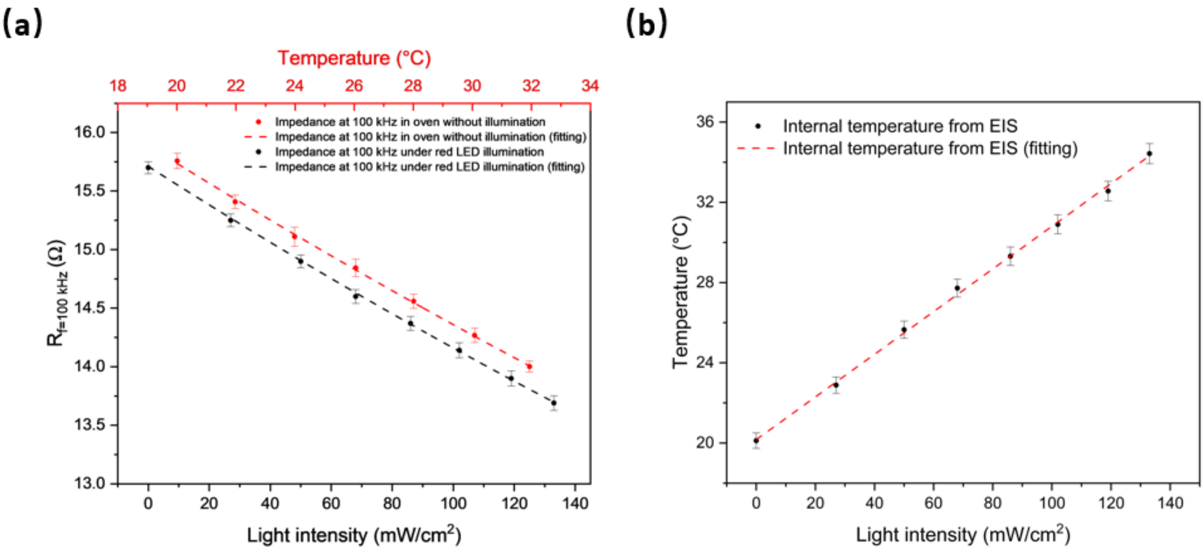

Figure S12. *(a) Impedance at 100 kHz as a function of light intensity (Red LED) and measured oven temperature. (fit: Arrhenius + R) (b) Estimated internal temperature as a function of light intensity (red LED).*

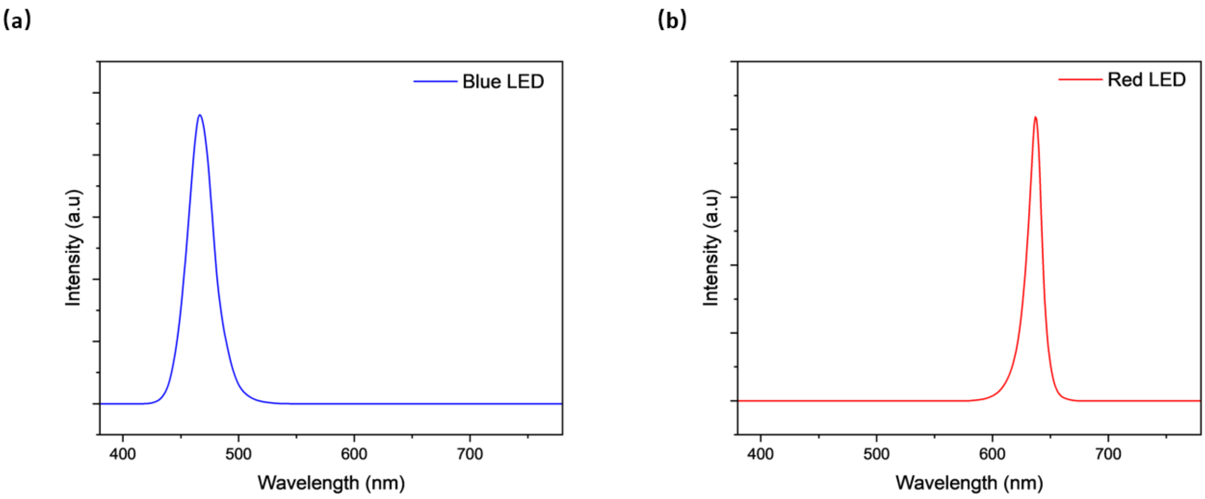

Figure S13. *Spectrum of the (a) blue and (b) red LED.*

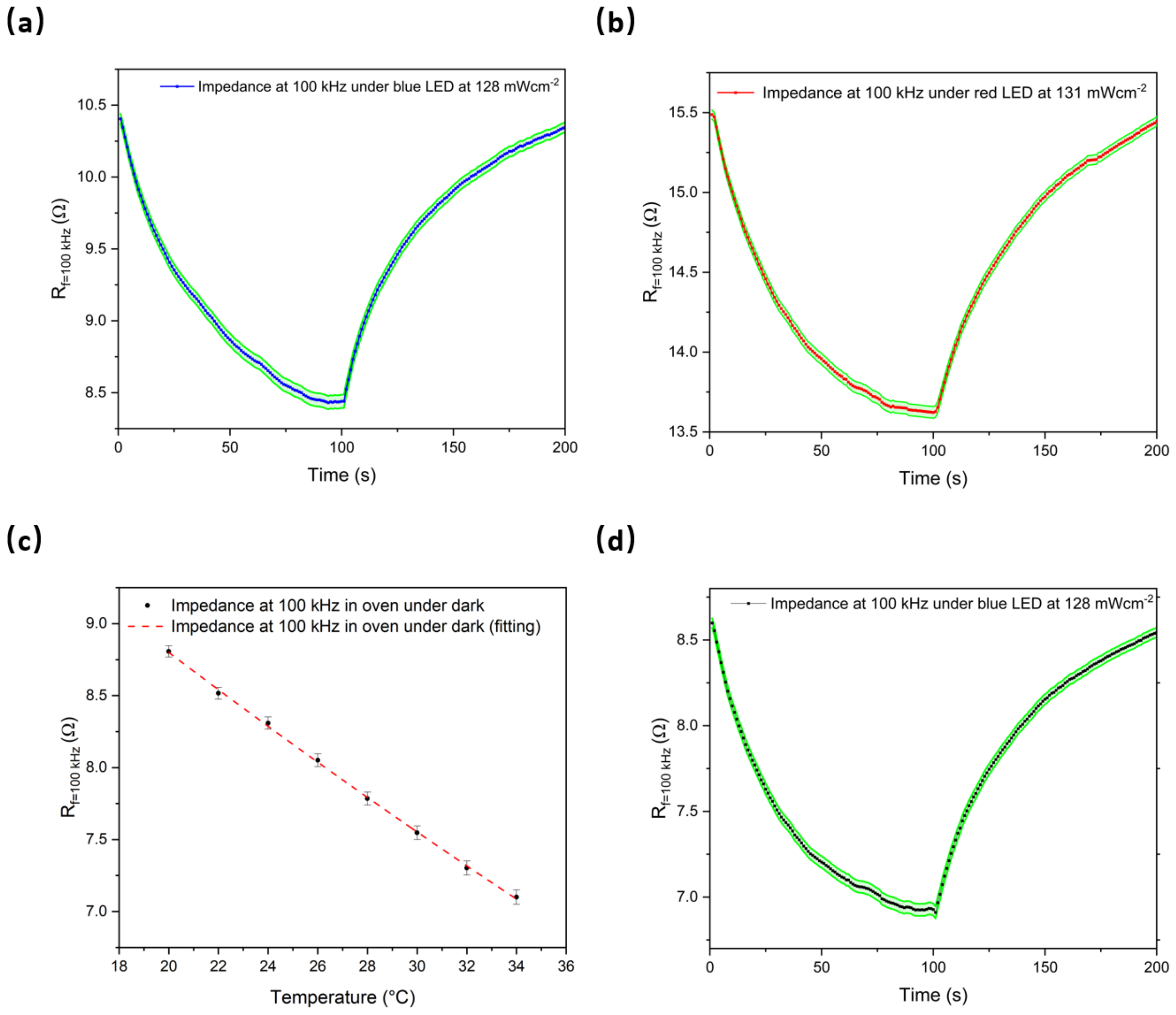

Figure S14. *(a) The impedance at 100 kHz during a continuous light-on/off cycle for blue LED at 128 mWcm^{-2}. (b) The impedance at 100 kHz during a continuous light-on/off cycle for red LED at 131 mWcm^{-2}. (c) Impedance at 100 kHz as a function of temperature for CF/Li half-cell. (fit: Arrhenius + R) (d) The impedance at 100 kHz during a continuous light-on/off cycle for blue LED at 128 mWcm^{-2} with CF/Li half-cell.*

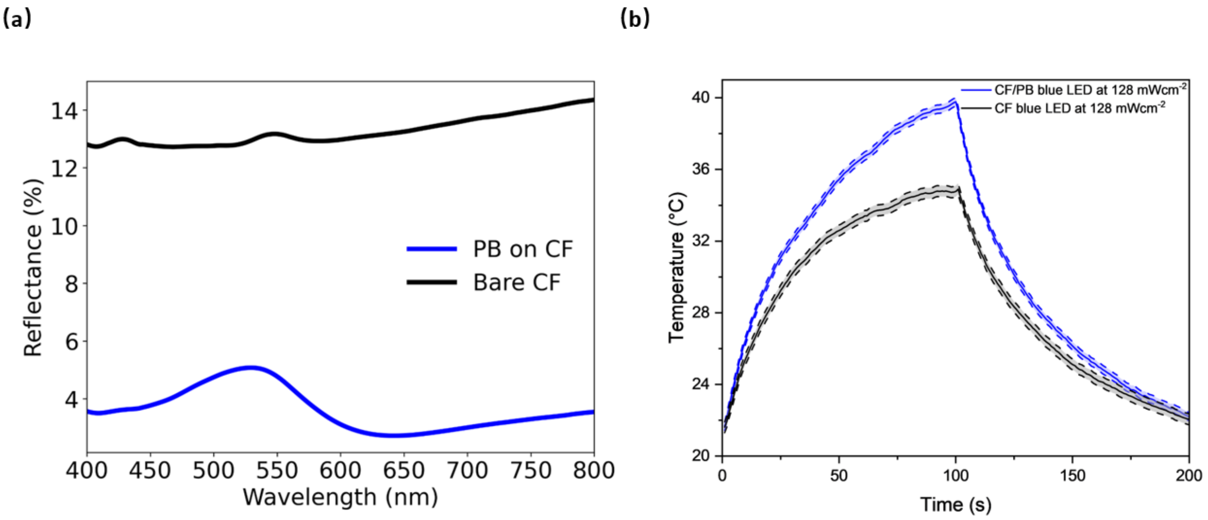

Figure S15. *(a) Reflectance spectra of bare CF and CF/PB electrode. (b) Heating and cooling curves of bare CF and CF/PB electrodes under blue LED.*

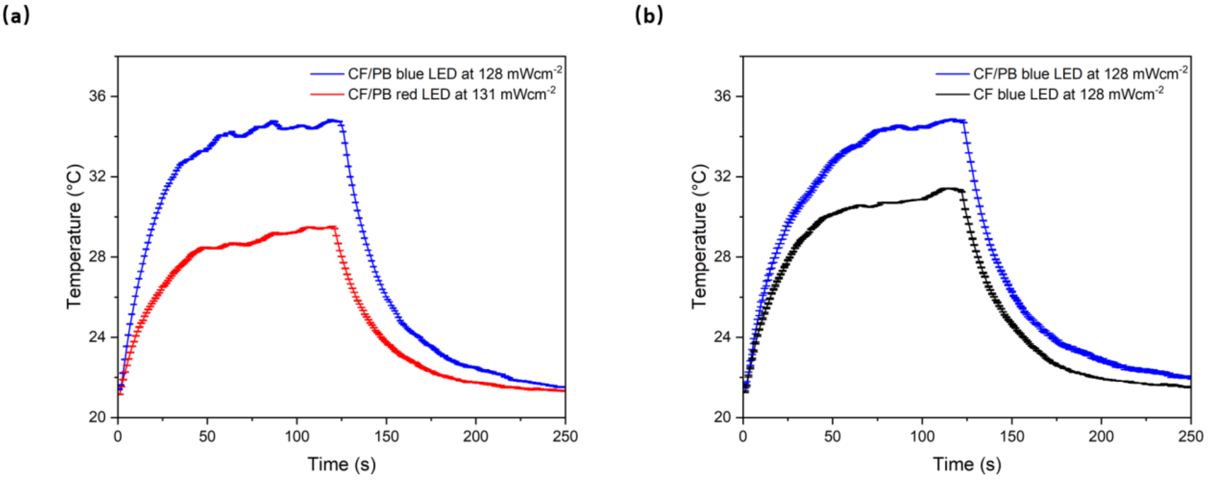

Figure S16. *(a) Heating and cooling curves of CF/PB under blue and red LED. (b) Heating and cooling curves of bare CF and CF/PB electrodes under blue LED.* ***(****Both were measured from a temperature probe)*

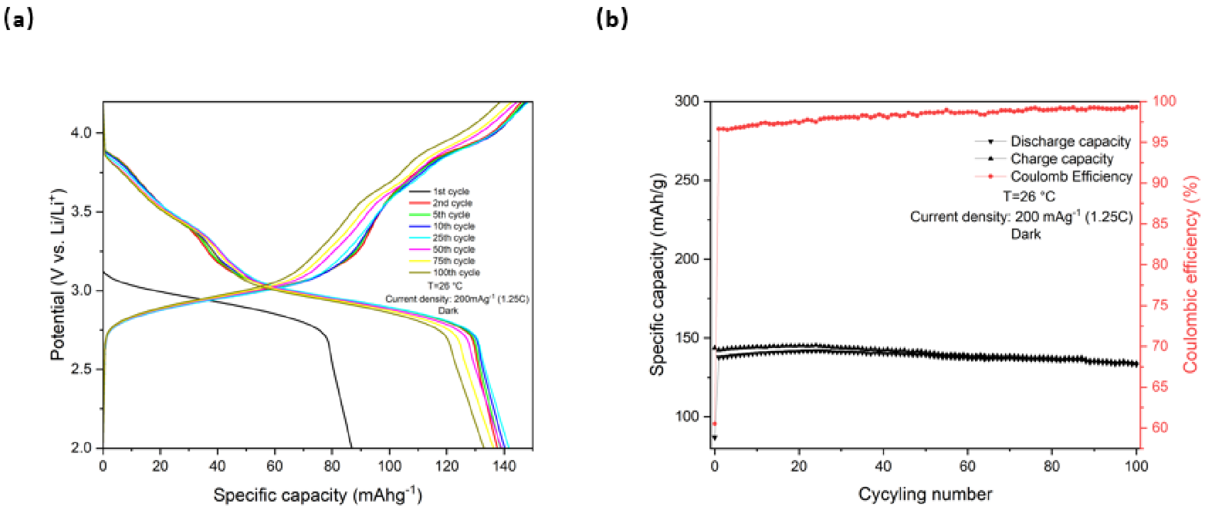

Figure S17. *(a) Galvanostatic charge-discharge curves at the specific current density of 200 mA/g under dark conditions at 26 °C at selected cycles. (b) Long-term cycling stability of the photo-LIBs under dark conditions at 26 °C at 200mAg^{-1}.*

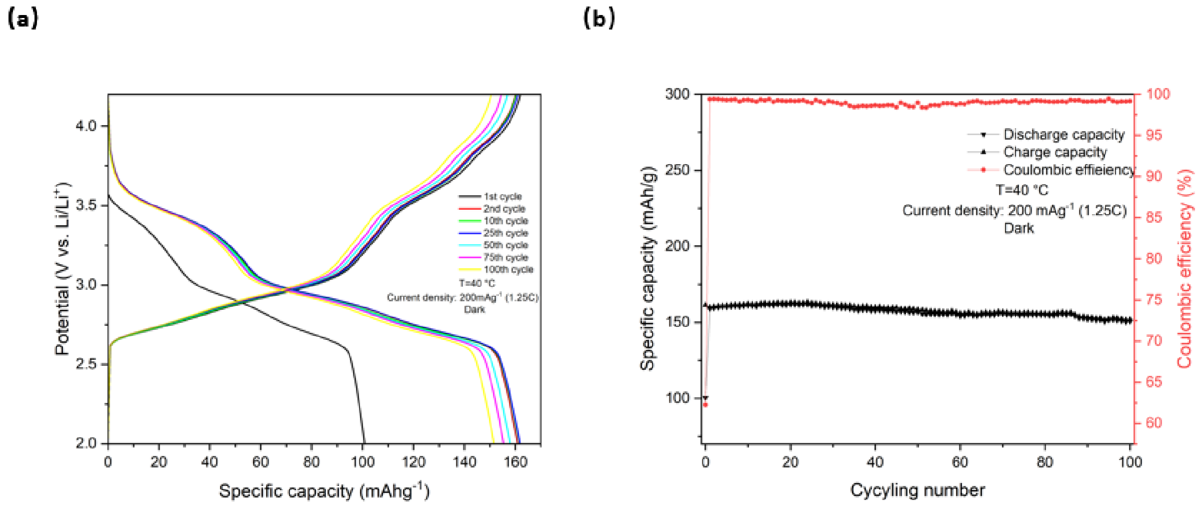

Figure S18. *(a) Galvanostatic charge-discharge curves at the specific current density of 200 mA/g under dark conditions at 40 °C at selected cycles. (b) Long-term cycling stability of the photo-LIBs under dark conditions at 40 °C at 200mAg^{-1}.*

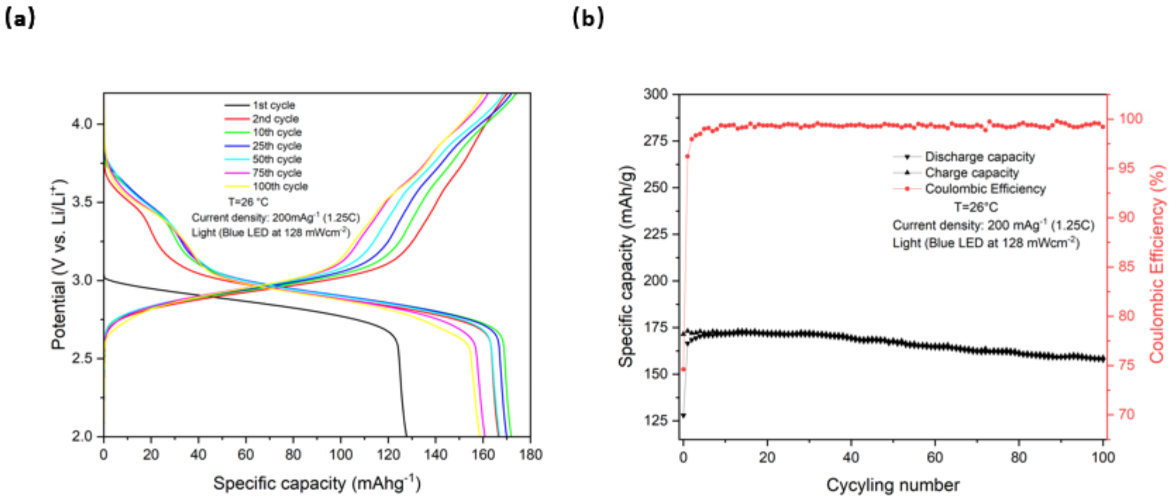

Figure S19. *(a) Galvanostatic charge-discharge curves at the specific current density of 200 mA/g under light conditions at 26 °C at selected cycles. (b) Long-term cycling stability of the photo-LIBs under light conditions at 26 °C at 200mAg*$^{-1}$*.*

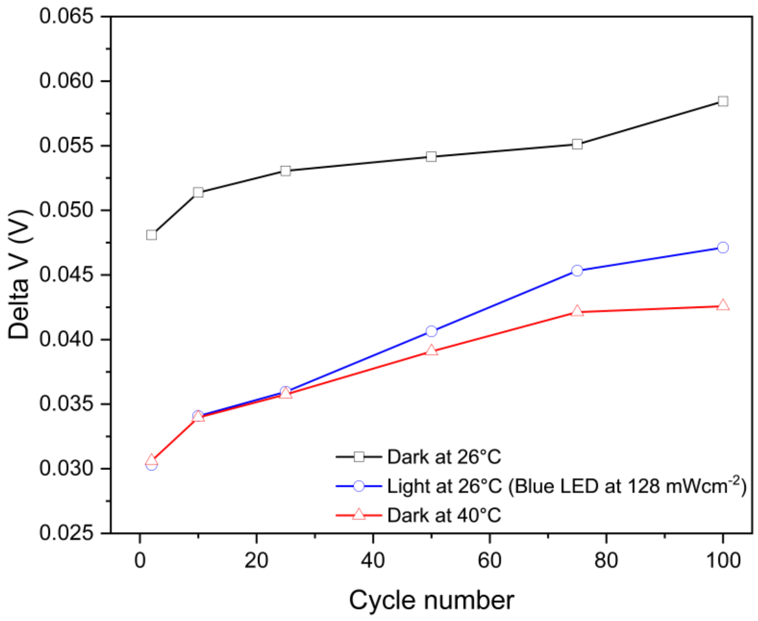

Figure S20. *The differences between nominal charge and discharge voltage (delta V) as a function of cycle number under Dark at 26°C, Dark at 40°C and Light at 26°C.*

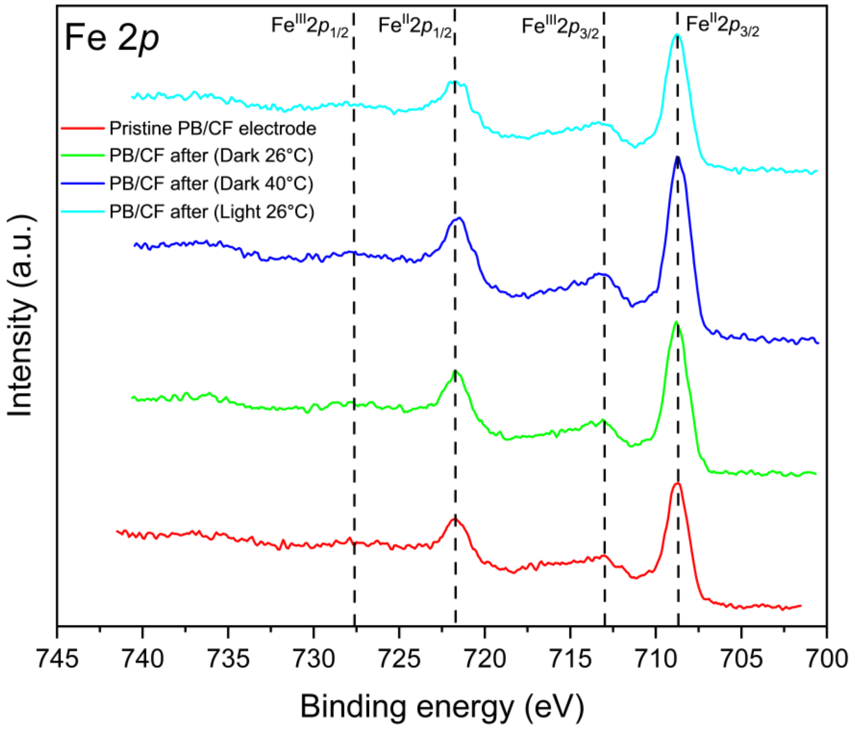

Figure S21. *XPS spectra of the characteristic Fe 2P peaks under Dark at 26°C, Dark at 40°C and Light at 26°C before and after cycling.*

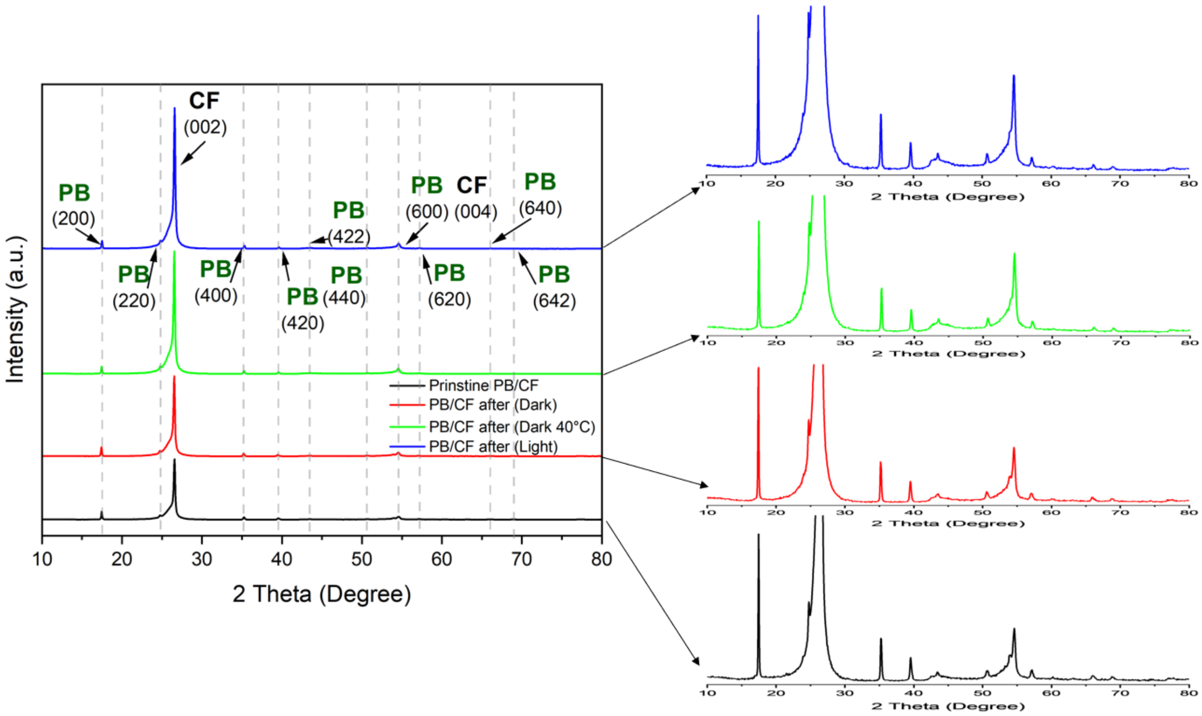

Figure S22. *XRD pattern of PB/CF electrodes under Dark at 26°C, Dark at 40°C and Light at 26°C before and after cycling.*
